# Identification and measurement of intensive economic growth in a Roman imperial province

**DOI:** 10.1126/sciadv.adk5517

**Published:** 2024-07-05

**Authors:** Scott G. Ortman, José Lobo, Lisa Lodwick, Rob Wiseman, Olivia Bulik, Victoria Harbison, Luís M. A. Bettencourt

**Affiliations:** ^1^ Department of Anthropology, University of Colorado Boulder, Boulder, CO, USA.; ^2^ Institute of Behavioral Science, University of Colorado Boulder, Boulder, CO, USA.; ^3^ Santa Fe Institute, Santa Fe, NM, USA.; ^4^ School of Sustainability, College of Global Futures, Arizona State University, Tempe, AZ, USA.; ^5^ Department of Archaeology, University of Cambridge, Cambridge, UK.; ^6^ McDonald Institute for Archaeological Research, University of Cambridge, Cambridge, UK.; ^7^ Department of Ecology and Evolution, University of Chicago, Chicago, IL, USA.

## Abstract

A key question in economic history is the degree to which preindustrial economies could generate sustained increases in per capita productivity. Previous studies suggest that, in many preindustrial contexts, growth was primarily a consequence of agglomeration. Here, we examine evidence for three different socioeconomic rates that are available from the archaeological record for Roman Britain. We find that all three measures show increasing returns to scale with settlement population, with a common elasticity that is consistent with the expectation from settlement scaling theory. We also identify a pattern of increase in baseline rates, similar to that observed in contemporary societies, suggesting that this economy did generate modest levels of per capita productivity growth over a four-century period. Last, we suggest that the observed growth is attributable to changes in transportation costs and to institutions and technologies related to socioeconomic interchange. These findings reinforce the view that differences between ancient and contemporary economies are more a matter of degree than kind.

## Teaser

Archaeological evidence shows that preindustrial societies could generate sustained increases in per capita productivity.

## INTRODUCTION

Economic growth is most simply defined as a sustained increase in a society’s production of goods, services, and information. The phenomenon has long been recognized as a consequence of two distinct processes: intensive growth, driven by improvements in the productivity and manner of input utilization, and extensive growth, driven by an expansion of the quantity of inputs (*1*–*4*). A well-established research tradition attributes intensive growth primarily to technological progress (*5*–*9*), although this should be interpreted broadly to include changes in culture and social institutions (*10*). Extensive growth, in contrast, is primarily a consequence of increasing the size of the labor force (*11*) through population growth, political integration, and/or an expanding market. The spatial concentration of population also facilitates a form of extensive growth known as Smithian growth by stimulating an expansion in the division of labor and facilitating knowledge spillovers (*2*, *12*–*14*). Distinguishing these different forms of growth in the empirical record has proven to be challenging and even a matter of controversy (*15*).

Settlement scaling theory (SST) provides a novel way to approach “growth accounting,” disentangling the effects of scale changes (such as population size) from effects generated by changes in agglomeration and technology and without making modeling assumptions that may or may not be appropriate conceptually, contextually, or empirically. Consequently, SST can be used to identify and distinguish the empirical signatures of various growth processes even in premodern, nonmarket contexts (*16*–*20*). The approach focuses on the relationship between the populations of “settlements,” which represent strongly interacting spatially embedded social networks and aggregate socioeconomic measures for these same spatial units. Scaling relationships express the basic insight that the size of a socioeconomic system is a crucial determinant of that system’s performance (*21*–*27*).

In this framework, the term extensive growth is used to refer to increases in per capita productivity driven by agglomeration effects, including the Smithian mechanism, which connects specialization and the division of labor to the size of local markets. This is related to simple demographic growth, but in addition to the raw number of people in a system, it also incorporates the distribution of population across settlements in that system. Intensive growth, in contrast, is used to refer to increases in per capita productivity driven by other, nonrival and nonexcludable factors, including social institutions and technologies. The key insight is that productivity gains can occur through agglomeration effects (which include specialization gains) as well as processes of institutional and technological change. We reserve the term intensive growth for only the second set of processes, acknowledging that productivity gains can also occur through population growth and agglomeration.

**Fig. 1.**
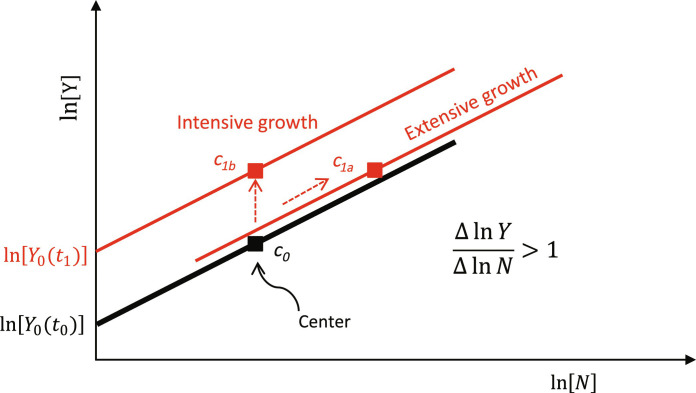
Economic growth in a settlement system. The black line represents the relationship between population and outputs at time *t*_0_, and the red lines represent possible relationships at time *t*_1_. Point *c* represents the center of the data (〈ln *N_t_*〉, 〈ln *Y_t_*〉, with brackets representing the mean of the respective data series). Because socioeconomic quantities are typically distributed log-normally, the center of the log-transformed data is the most reasonable summary. Two types of changes can occur over time: (i) The size (or scale) of the system can increase, shifting the center along the scaling relationship to point *c*_1*a*_; and (ii) the height of the scaling relationship can change, raising the center to point *c*_1*b*_. Note that, in the diagram, the resulting aggregate output at time *t*_1_ is the same, but in (1a), the increase is due to changes in agglomeration, whereas in (1b), it is due to changes in baseline productivity so that per capita productivity (〈ln *Y_t_*〉/〈ln *N_t_*〉) will be higher in (1b) than in (1a). However, per capita productivity in (1a) can still be higher than it was at *t*_0_ if the scaling relationship has a slope (∆ ln *Y*/∆ ln *N*) that is greater than 1.

Figure 1 illustrates how the changing relationship between population and outputs across settlements can be used to disentangle these different forms of growth. The key observation is that the slope of the relationship between log-transformed population and output measures is typically greater than 1 and is consistent across settlement sizes and across systems, even as the baseline level of the relationship may change over time (*4*, *28*–*30*). As a result, increases in per capita outputs can occur through either the demographic processes of agglomeration, which shift the center of the system along the scaling relation, or social or technological changes that increase the baseline productivity of human labor across the settlement system, thus raising the height of the overall scaling relationship. Scaling analysis has been used to examine the economies of a wide range of societies ranging from the contemporary urban systems of the United States, China, and India to a variety of ancient societies known through archaeology. These studies have shown that, in contemporary societies, both intensive and extensive growth typically occur simultaneously (*18*, *19*), and intensive growth is becoming more important as population growth decreases. However, in archaeological data, the general pattern in studies to date has been one of long-term stability in the height of scaling relationships, such that any changes in proxy measures of output per capita are attributable almost entirely to agglomeration effects related to changes in the size distribution of settlements (*16*, *17*, *31*, *32*). This contrast between ancient and contemporary systems raises several questions. Are the observed differences due to the limited precision of archaeological data or to methods that wash out the potential for a changing scaling relationship? Could it be that commonly available archaeological proxies for socioeconomic outputs are insensitive to the effects of intensive growth? Or did premodern economies not generate or sustain intensive growth at rates fast enough to be seen in the data?

Recent examinations of ancient economies in the Mediterranean and Aegean world, using newer lines of evidence, have shown that economic change did occur in past societies, leading to changes in living standards and productivity (at least for some segments of the population) (*33*–*38*). It can thus be concluded that economic growth of some sort did occur in the preindustrial world. Whether it was extensive or intensive, sustainable or unsustainable, and whether the nature and drivers of economic change in ancient societies were similar to or different from those of “modern” societies are thus matters for empirical investigation (*38*–*40*). Here, we contribute to the understanding of economic growth in premodern societies by investigating the changing relationship between archaeological proxies for settlement population and socioeconomic rates in Roman Britain. We show that social arrangements invoked as explanations for modern episodes of economic growth also occurred in Roman Britain, and we argue that the presence of such drivers is not conceptually or empirically incompatible with slower growth rates prior to the onset of the fossil fuel economy.

There are several reasons why Roman Britain is a useful context for such a study. First, the Roman conquest led to the incorporation of portions of Britain into the Imperial Roman economy, which one might expect to have initiated a process of “globalization” and technological catch-up growth on the island. Second, the Romans founded the first cities and towns in Britain, and additional towns developed spontaneously in subsequent centuries, suggesting that the population became increasingly agglomerated over time. Third, a variety of lines of evidence suggest improvements in transport technology (roads, wheeled vehicles, and stronger draft animals), economic institutions (coinage and rule of law), agricultural land and labor productivity (ploughs, draft animals, mills, and corn dryers), and housing (stone masonry, in-floor heating, and tile roofs), all of which are consistent with economic growth. Last, Roman period remains have long been a focus of attention in British archaeology, and this has only accelerated in recent decades through developer-funded excavation, leading to an extensive and robust database for investigating processes of economic change using archaeological data.

We first present a formal framework that (i) derives the consistent elasticity of scaling relationships from first principles and (ii) allows one to distinguish between extensive and intensive growth through scaling analysis. Then, we provide some background on the Roman economy and a description of the dataset compiled here to examine economic change over time. Third, we estimate the relationship between population size and three distinct socioeconomic measures for four time steps spanning the Late Iron Age through the end of the Roman period, finding evidence of intensive growth in this preindustrial economy. Last, we return to the formal framework and additional related studies to begin the process of accounting for the observed intensive growth, and we consider the implications of our results for a general framework that unifies the study of economic change across time, geographies, and levels of development.

## DISTINGUISHING INTENSIVE VERSUS EXTENSIVE GROWTH

The theoretical framework known as SST provides an analytical procedure for distinguishing (social or physical) technology-driven from agglomeration-driven changes in per capita productivity. The basic models and empirical evidence in support of SST have been presented in previous publications (*4*, *17*–*19*, *28*, *41*, *42*). Here, we focus on its connection with growth in material outputs.

A fundamental axiom of SST is that per capita productivity is proportional to the average interaction rate among individuals (the degree *k* of an individual’s undirected socioeconomic network), given the frictional effects of distance. This notion, that increasing productivity derives in part from the concentration and intensification of social interchange, is the basic idea behind economic models of agglomeration effects, from lowered transaction costs to increased complementarities in production to knowledge spillovers (*1*, *43*–*45*). Together with the assumption that humans mix socially as much as possible, given spatial constraints, the overall output of a strongly interacting population in a settlement can be written as

$$Y_{i}\left(N_{i},t\right)=G\left(t\right)\frac{{\left[N_{i}\left(t\right)\right]}^{2}}{{A_{n}}_{i}\left(t\right)}\times e^{\varepsilon_{i}\left(t\right)}$$ (1)

where *Y_i_*(*N_i_*, *t*) is the aggregate output (a socioeconomic rate) of a given settlement *Y_i_* with resident population *N_i_* at time *t*, [*N_i_*(*t*)]^2^ ≈ *N_i_*(*t*) × [*N_i_*(*t*) − 1] is the total number of links in the undirected social network represented by that population, *A_ni_*(*t*) is the spatial extent (area) over which interactions occur in that population, *G*(*t*) represents the productivity of social interchange (average output per interaction), and the stochastic term *e*^ε*_i_*(*t*)^ captures the range of influences unique to each location that lead to a deviation of productivity in any given settlement from the average expectation. Statistically, the ε(*t*) term accounts for deviations in each settlement from the expected (power-function) scaling relationship. We represent this deviation as an exponential so that it will take the form of a Gaussian white noise, generating typically observed lognormal statistics for *Y_i_*(*N_i_*, *t*) (*19*).

Equation 1 can be simplified by expressing *A_ni_*(*t*)—the area of the settlement over which people interact—in terms of *N_i_*(*t*). We accomplish this in several steps (subscripts, errors, and time are omitted below to simplify the notation). First, we consider the balance of costs and benefits for individuals when they interact from the perspective of a spatial equilibrium whereby the benefits and costs of locational activities and choices balance each other (*46*). In the case of a settlement without deliberately constructed infrastructure, the cost for a person to interact with others is largely set by the energetic cost of traversing the maximum distance between individuals, which is given by *c* = μ*A*^1/2^ (where μ is the cost of movement and *A* is the circumscribing area); and the benefit of the resulting interchange is given by *y* = *GN*/*A*, following from Eq. 1. There is an implicit assumption here that human effort has limits and tends to balance benefits and costs. In the context of moving in physical space, this implies that individuals access, or explore, only those portions of the space in which their social interactions are embedded (*47*). Setting *c* = *y* (balancing costs with benefits) and simplifying, one arrives at

$$A\left(N\right)={\left(G/\mu \right)}^{2/3}N^{2/3}=aN^{2/3}$$ (2)

where *a* = (*G*/μ)^2/3^. Thus, the area circumscribing a settlement grows proportionately to the settlement population raised to the 2/3 power, such that larger settlements become progressively denser on average. Note also that the prefactor *a* of this relationship varies in accordance with the productivity of social interchange and with transportation costs but is independent of population.

Second, we consider how the interaction area itself changes as settlements become larger and denser. With densification, social interchanges increasingly occur through movement along an access network of roads, paths, and other public spaces as opposed to freely chosen straight paths. As in spatial economics, we assume that expansion of the infrastructure needed to facilitate interaction responds to the expansion of concentrated population (*48*). The space devoted to the access network ρ is thus added to the settlement in accordance with the current population density, ρ = (*N*/*A*)^−1/2^, such that the total area of the access network *A_n_* = *N*ρ = *A*^1/2^*N*^1/2^. Substituting *aN*^2/3^ for *A* in this equation, based on Eq. 2, then leads to (with subscripts and time added back in)

$${A_{n}}_{i}\left(t\right)=a{\left(t\right)}^{1/2}N_{i}{\left(t\right)}^{5/6}$$ (3)

Equation 3 indicates that the area over which interaction occurs in a settlement grows proportionately to the settlement population raised to the 5/6 power. We can now substitute Eq. 3 into Eq. 1 and simplify, leading to

$$Y_{i}\left(N_{i},t\right)=Y_{0}\left(t\right){\left[N_{i}\left(t\right)\right]}^{\beta }\times e^{\varepsilon_{i}\left(t\right)}$$ (4)

with β = 2 − 5/6 = 7/6, and $Y_{0}\left(t\right)={G_{t}}^{2/3}/{\mu_{t}}^{1/3}$ . Note that Eq. 4 is a type of production function in which the output of a city or settlement depends on the labor input (population size) and there is a level of productivity characteristic of the settlement system (*44*). This system-wide productivity level stems from the settlements constituting a system sharing social, technological, cultural, and political features; which is to say, they share nonrival and nonexcludable factors (*1*).

Equation (4) states that, as settlements increase in population, their average aggregate socioeconomic rates grow proportionately to the population raised to the β = 7/6 = 1.167 power. It also suggests that the baseline level of productivity across all settlements, *Y*_0_(*t*), is a function of the strength and productivity of social interactions, *G_t_*, involving factors such as organization, technology, and institutions, and the energetic cost of movement μ*_t_*, both at time *t*. Last, it suggests per capita rates are given by

$$y_{i}\left(N_{i},t\right)=Y_{0}\left(t\right){\left[N_{i}\left(t\right)\right]}^{\delta }\times e^{\varepsilon_{i}\left(t\right)}$$ (5)

which implies that increasing per capita productivity is proportional to population raised to the δ = β − 1 = 1/6 = 0.167 power. Together, Eqs. 4 and 5 imply that there are increasing returns to scale such that more populous settlements are more productive per capita simply due to the network effects of individuals interacting regularly in space. In (*17*, *49*, *50*), we show that this framework can also be extended to capture the effects of increasing connectivity between individuals for specialization and the division of labor, leading to the form of growth we refer to as extensive or Smithian growth.

We next show that this framework captures the overall pattern of economic growth in a society. We first take the natural logarithm of Eq. 4 to express it as a linear function

$$\text{ln}\left[Y_{i}\left(N_{i},t\right)\right]=\text{ln}\left[Y_{0}\left(t\right)\right]+\mathrm{\beta ln}\left[N_{i}\left(t\right)\right]+\varepsilon_{i}\left(t\right)$$ (6)

Then, we express this result in terms of the ensemble average across all settlements (*19*)

$$\langle \text{ln}\left[Y\left(t\right)\right]\rangle =\text{ln}\left[Y_{0}\left(t\right)\right]+\beta \langle \text{ln}\left[N\left(t\right)\right]\rangle$$ (7)

where the ensemble averages are defined as

$$\langle \text{ln}\left[Y\left(t\right)\right]\rangle =\frac{1}{N_{s}}\sum_{i=1}^{N_{s}}\text{ln}Y_{i}\left(N_{i},t\right)$$ (8a)

$$\langle \text{ln}\left[N\left(t\right)\right]\rangle =\frac{1}{N_{s}}\sum_{i=1}^{N_{s}}\text{ln}N_{i}\left(t\right)$$ (8b)

and where *N_s_* refers to the total number of settlements. Because, by definition, 〈ε*_i_*(*t*)〉 = 0, it can be dropped from the ensemble average. Equation 7 indicates that the average log-output of settlements in a system at a particular time is the sum of log-baseline productivity (which incorporates nonrivalrous technology) and the average log-population of settlements multiplied by the scaling exponent β = 7/6. One can also think of Eq. 7 as representing the center of the data in Fig. 1, the average relation of which is given by Eq. 6 (minus the error term), a line with slope β and intercept ln[*Y*_0_(*t*)]. Note also that Eq. 8a implies that the total output of the society is given by ln*Y*(*t*) = *N_s_* × 〈ln [*Y*(*t*)]〉.

We can differentiate both sides of Eq. 7 to convert it to a growth equation

$$\frac{d\langle \text{ln}Y\rangle }{\mathrm{dt}}=\frac{d\left(\text{ln}Y_{0}\right)}{\mathrm{dt}}+\beta \frac{d\langle \text{ln}N\rangle }{\mathrm{dt}}\to \gamma_{\langle Y\rangle }=\gamma_{Y_{0}}+\beta \gamma_{\langle N\rangle }$$ (9)

Equation 9 is in effect a sort of growth accounting (*51*, *52*), which states that the total growth rate of mean settlement output is the sum of the growth rate of baseline productivity [the *Y*_0_ term, akin to total factor productivity (TFP)] and the growth rate of the mean settlement population multiplied by β = 7/6. According to Eq. 9, the overall growth in material output can be decomposed into growth in baseline productivity γ_*Y*_0__, which is driven by a variety of factors that affect movement costs and interaction benefits, and growth due to the agglomeration effects generated by an increase in population size, βγ_〈*N*〉_.

An important aspect of this framework is that, because the slope of the relationship between population and output is consistent and scale free, it is possible to estimate ln[*Y*_0_(*t*)] and β even using a nonrandom sample of observations for which the coordinates of the center for the sample (〈 ln [*N*(*t*)]〉, 〈ln [*Y*(*t*)]〉) do not provide a reasonable estimate of the center of the entire system. This is because the scaling prefactor is systemic, i.e., common to all settlements, and can be detected by sampling from the set of all settlements, and the scaling exponent is also a collective property of a settlement system that can be estimated from any sample of places that vary in scale. So long as the intercept of the scaling relation ln[*Y*_0_(*t*)] representing baseline productivity can be estimated, analysis of changes in this intercept over time will reveal patterns of intensive growth. We use this useful feature of SST here, in a situation where the data represent a nonrandom sample of partially excavated archaeological sites.

## THE ROMAN ECONOMY AND ROMAN BRITAIN

For many decades, ancient historians maintained that ancient economies were static, such that all but a small landowning elite lived at near-subsistence levels, and increasing population was generally detrimental to living standards for most people (*53*). However, in recent decades, researchers have reexamined ancient written sources and archaeological evidence in arguing that the Roman world was relatively prosperous and dynamical (*34*, *39*, *54*–*62*). Following this recognition, debate has turned to whether improvements in the population’s material well-being were a simple by-product of population growth or whether per capita productivity also increased (*38*, *63*–*69*).

A variety of lines of evidence suggest that both extensive and intensive growth took place in the centuries following the Roman conquest of Britain. Primary towns with specific administrative functions were established immediately following the conquest, mostly at locations of Late Iron Age oppida and subsequent Roman military installations; and additional small towns and roadside settlements developed in subsequent centuries at crossroads and along major routes of travel, indicating substantial increases in the “urbanized” population (*70*). Studies of rural sites (mostly farmsteads and villages) in combination with urban settlements suggest that the fraction of population that lived in towns increased from about 0% to 10 to 20% during the Roman period (*71*). Whether the overall population increased, decreased, or remained stable depends on changes in the sizes and densities of urban sites, which remain poorly understood. There does seem to be a decline in the number of inhabited rural sites over the course of the Roman period, but there is also evidence for increasing farm productivity, which could have supported a higher urbanization rate (*72*–*74*). Other evidence, including a shift from timber to stone masonry buildings, increasing occurrence of mosaics and in-floor (hypocaust) heating systems, increasing numbers of metal artifacts, increasingly standardized manufacture of household goods, evidence of greater manufacturing specialization, improvements in farm productivity, and construction of a province-wide road network to which the population increasingly oriented, all support the conclusion that material living standards increased for at least some people during the Roman period (*65*, *75*–*77*). Together, these empirical observations suggest that, during the Roman period, the economy of Britain experienced substantial per capita growth (i.e., increases in productivity and consumption), not just increases in gross output. Here, we seek to quantify this growth by examining proxy measures of socioeconomic rates available from the archaeological record.

## MATERIALS AND METHODS

**Fig. 2.**
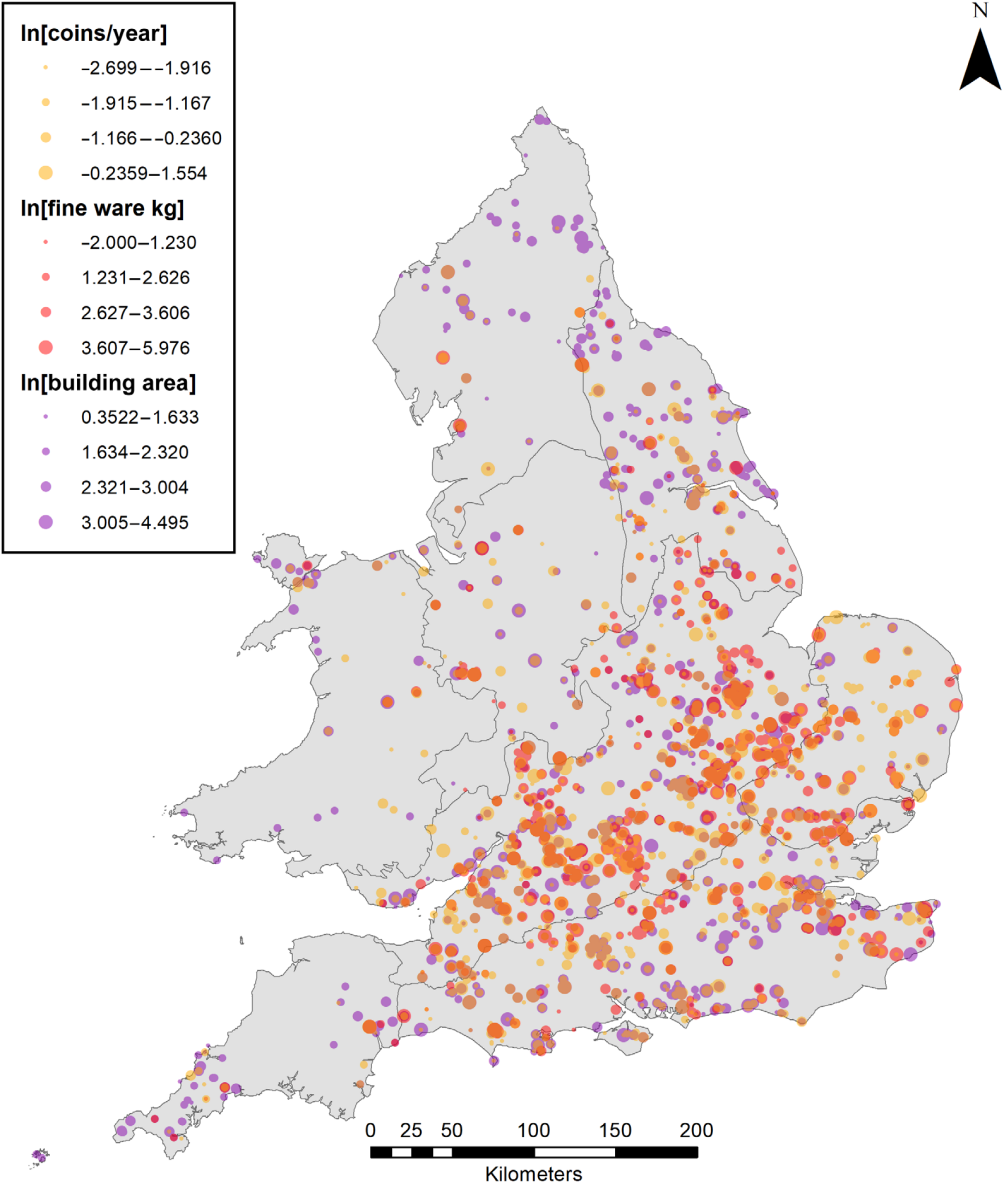
Spatial representation of the dataset. Polygons are regions of Britannia defined by the Rural Roman Settlement Project. Symbol colors and sizes represent the sample sizes of coins (count/year), fine wares (kilograms), and building areas (square meters). All settlements are associated with building counts.

The data used in this study derive from archaeological excavations at Romano-British settlements in England and Wales. In the Supplementary Materials, we provide background on our data sources and the resulting database. Each record represents an excavation “site” encompassing all or part of a Roman era settlement. For this study, we focus on the coins, pottery, and buildings from these excavations. For each settlement, we constructed measures of coin loss, fine pottery consumption, housing consumption, and the associated population. Figure 2 displays the dataset on a map. The procedures we used to construct these measures are described below.

### Coin loss

Excavation reports, and the source databases from which we derived our dataset, generally distinguish “site finds” representing incidental loss of small change from “hoards” representing intentional deposition of wealth for safekeeping. We follow standard practice and focus on site finds (*78*) because one would expect these to bear a more consistent relationship to the purchasing power of associated residents than hoards, which represent intentional deposition of high-value coins in hidden locations during periods of instability, followed by chance rediscovery by archaeologists (*79*–*81*), leading to extreme sampling issues (*82*). The date of manufacture of a coin can often be determined precisely through knowledge of the reigns of the rulers depicted on them, but the issue date distribution of coins from an excavation is not as useful for tracking economic fortunes as one might assume. Studies of coin hoards show that most coins circulated for several years before entering the archaeological record (*83*), and lost coins could also be redeposited through later earthmoving events. In addition, studies of site finds generally find strong fluctuations in the relative abundance of coins by issue date that are attributable to short-term monetary and political factors, which affected the age distribution of coins in circulation more so than the total value of those coins (*84*). As a result, the specific date distribution in site finds reflect monetary supply trends more so than the specific economic fortunes of the site inhabitants.

We are interested in relationships between the coins and the buildings found in an excavated area, and any method of apportioning coins to time periods that was independent of the process used to apportion buildings to time periods would destroy any such relationships. Building construction and abandonment dates are often based on the most recent coins found stratigraphically immediately beneath and above floors and foundations, respectively. As a result, the issue date distribution of the coins from an excavation bears no necessary relationship to the date distribution of the associated buildings or the actual rates of coin loss by the residents during the occupation (see the Supplementary Materials for additional discussion). Given this, we proceed simply and divide the total number of coins (excluding hoards) by the duration of occupation of each excavated area to calculate the average coin loss rate per year of occupation for that area. To estimate the number of people associated with these coins, we use the total number of buildings encountered within the excavated area. Many building types were clearly not residences, but studies of recent settlement systems that connect the built environment to census data show that raw building counts are strongly correlated with population (*85*), and archaeological studies have also found that building count is a useful population proxy (*86*). Thus, while this measure is not perfect, it does have empirical support. In sites where there are superimposed buildings, each building is counted separately. This is reasonable because such sites have longer overall durations, and as a result, the coin accumulation should be divided by a larger number as well.

Last, we assign the coins from each excavation to one of four time periods based on the midpoint of the beginning and end dates of the occupation, as determined by the excavators using whatever lines of evidence were available to them. When there is more than one excavation from the same settlement, we average the values for the excavations assigned to each period to estimate the coin loss rate and population for each settlement and period. The assumptions being made here are that lost and/or discarded coins represent small change, are proportional to the number and intrinsic value of the coins that circulated among the people who lived in a settlement, and are proportional to the economic conditions experienced by these residents (*79*, *87*).

### Pottery accumulation

Problems with the recording of Roman pottery in Britain are well documented (*88*), and putting this material into a usable order involved a separate subproject (*89*). In brief, we sourced the original excavation reports for sites with quantified assemblages of at least 500 sherds or 4 kg of pottery. From these reports, we compiled quantities of each of the pottery fabrics reported. Fabrics are important as they can be used to isolate the industry that produced the pottery as well as the broad uses to which pottery was put. Finer fabrics were used to make table wares; coarser fabrics were used for making storage vessels and cooking pots. We also recorded the presence of amphorae (for transporting goods), flagons (for storing liquids), and mortaria (for preparing food). We compiled data for 775 excavations covering 652 separate settlements. As part of this process, we determined the pottery class (Samian, coarse ware, fine ware, mortaria, flagon, amphora, and storage vessel) for each entry, following the classification developed by Tyers (*90*). To harmonize the various methods of quantification, we prioritized weight and translated other forms of quantification into estimated weights. If weights were absent but counts were available, then we multiplied counts by the average sherd weight across the dataset (15 g) to convert these into estimated weights. Also, if only estimated vessel equivalents were available, then we treated these as counts and converted them to estimated weights as well.

We divide the total weight of fine wares by the total weight of coarse wares from each excavation to calculate a ratio representing the relative accumulation rate of fine wares. The logic of this measure is that coarse wares were used primarily for food storage and preparation. These activities should be proportional to population and time and inelastic with respect to economic development. In contrast, fine wares are used primarily in the context of socializing with food, and as a result, household inventories of fine wares, and use rates of fine wares, should increase with material living standards, leading to increases in their accumulation rate relative to that of coarse wares (*16*, *17*, *91*, *92*). Because this ratio already represents an average over the occupation span, there is no need to divide by the duration as was done for coins. However, it also represents the relative consumption rate of fine pottery per person-year. Thus, to convert it to an aggregate measure, we multiply this ratio by the total number of buildings encountered in the excavation. This converts the ratio from a per capita measure to an aggregate measure that is parallel to the coin loss rate. As with coins, we assign each excavation to a time period based on the midpoint of the date range determined by the excavators for the Roman occupation within that excavation area. Also as with coins, we average these values across excavations from each settlement and period to estimate the fine pottery consumption rate by settlement and period. Last, again as with coins, we use the total number of buildings encountered within the excavated area as a proxy for the number of people associated with this consumption rate.

### Building area

Several previous studies have shown that the area of a residential building is a good proxy for the productivity of the people who lived in that building (*32*, *33*, *93*–*101*). We therefore use the total area of residential buildings within an excavation area as a measure of the total economic output of the people represented by those buildings. In the Roman period, residential buildings took two basic forms: circular and rectangular. The source data files record the number of each that was fully or partially exposed by the excavation, along with the presence of other building types. We used this information, and the associated references, to consult the primary literature for each excavation that encountered buildings and measure the area of each building exposed in that excavation. Unlike coins and pottery, construction and abandonment dates can often be determined for individual buildings through stratigraphy and associated finds. Also, unlike coins and pottery, it is straightforward to apportion the buildings encountered in an excavation to time periods without destroying the relationship between housing consumption (residential building area) and population (residential building count). Thus, we assigned each building to one of our four periods based on the midpoint of its occupation dates, as presented in the excavation report, and then we grouped these together to determine the count and total area of residential buildings within an excavation area during each period. Each building was assigned to one and only one period. We estimated the area (including courtyards) of each building using dimensions provided in the reports or by measuring the perimeter of the walls from digitized plan drawings. When buildings continued beyond the boundary of the excavation, we included only those portions within the excavated area in the total building area calculation.

### Chronology

The goal of this analysis is to assess changing economic rates over time. Consequently, we needed to place the data into temporal groups. For this study we assigned materials to one of four time periods: Late Iron Age (approximately 200 BC to 50 AD), Early Roman (50 to 150 AD), Middle Roman (150 to 250 AD), and Late Roman (250 to 400 AD). Although these periods are labeled using round numbers, their boundaries correspond approximately to major events in the history of Roman Britain, including the Claudian invasion, the Antonine Plague, the “third century crisis,” and the departure of the Roman legions.

### Additional notes

It is important to be clear about exactly what is being measured and compared. SST focuses on relationships among extensive properties of entire settlements, but the three measures we examine are constructed with respect to an excavated area, and the measures themselves represent observations drawn from nonrandom samples of the total materials that exist at each settlement. As a result, the actual comparisons are between coin density per year, the density of fine ware per person-year, and the fraction of a settlement that is taken up by residences, for portions of settlements. Because most Romano-British settlements have poor surface visibility, the actual settlement size, and thus the fraction of the total represented by the excavated materials, is often only vaguely known. There are two reasons why this shortcoming can be overcome. First, scaling relations are, by definition, scale-free, and as a result, the parameters of the relation (the slope and intercept from a linear fit to log-transformed measures of population and a socioeconomic rate) can be estimated from a sample of settlements from a system. Second, all three measures we use are with respect to the same excavated area, so any scaling relationship between population density and output density will be equivalent to the relationship between population and output. Third, one of the most robust patterns in human settlement systems is that larger settlements are generally denser (*102*–*104*), so one would expect scaling relationships between population density and output density to be strongly correlated with relationships between population and output on this basis as well. Still, it is important to remember that settlement populations and material outputs are not being measured directly, so the center of the data in our analyses reflects the size distribution of excavation projects more so than the population distribution of settlements. As a result, these data only allow us to capture the intensive component of economic growth (change in the intercept of the scaling relation), as discussed above.

## RESULTS

We estimate parameters of the scaling relation between buildings and coins per year of occupation, and between buildings and the ratio of fine ware to coarse ware, for each of four chronological periods. This involves fitting a linear model to the two variables being compared, following transformation to natural logarithms. This is feasible because *Y*(*N*) = *Y*_0_*N*^β^ and log[*Y*(*N*)] = βlog[*N*] + log [*Y*_0_] are equivalent expressions, and the latter is a simple linear function, leading to a simple estimation problem for the parameters: The slope of the best-fit line for the log-transformed data (β) is also an estimate of the exponent of the scaling relation, and the *y* intercept of the best-fit line (log[*Y*_0_]) is an estimate of the logarithm of the prefactor (*Y*_0_) of the scaling relation. Because these data are noisy and the individual data points can be expected to exhibit sampling error, we bin the data, following log transformation, in 0.25 increments to facilitate assessment of the average relationships (*105*), and we estimate standard errors using the White correction for heteroskedasticity.

The SST framework presented above leads to two predictions for these analyses. First, it predicts that the slope of the relationship between log-transformed population and aggregate output measures β will be close to 7/6 in all cases where the dependent variable measures a socioeconomic rate (as opposed to an accumulated stock, which would be expected to have a steeper slope). Second, it predicts that, due to the increases in per capita productivity suggested by previous research on the Roman economy and Roman Britain in particular, the intercept of relationships between population and outputs, log[*Y*_0_], should increase over time, with any such increases being driven by changes in transport costs μ*_t_* and the strength and energetic productivity of social interchange deriving from social institutions and technology, *G_t_*, according to the relation $Y_{0}\left(t\right)={G_{t}}^{2/3}/{\mu_{t}}^{1/3}$.

### Coin loss

**Fig. 3.**
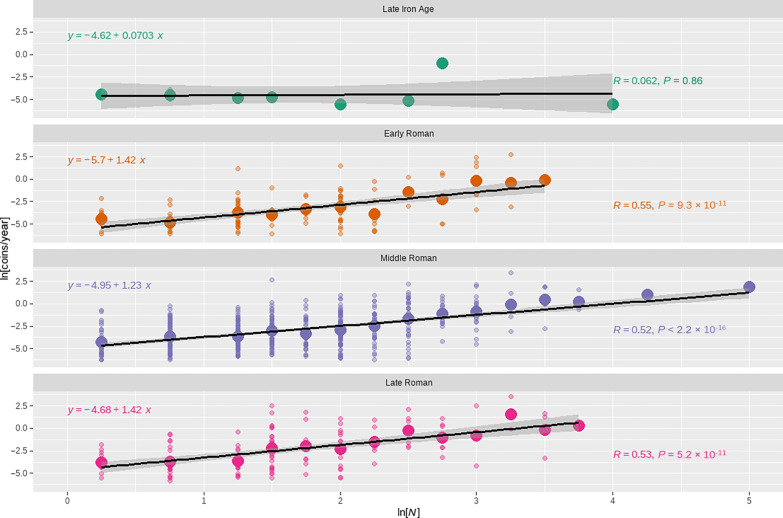
Relationship between coin loss rates and population over time. The data have been binned by building count, in 0.25 increments, following log transformation. Small open circles represent individual cases, and larger filled circles represent the mean value for each bin. The relationship between coin loss rate and population is not statistically significant for the Late Iron Age, the slope of the relationship is relatively consistent and greater than 1 across the Roman periods, and the height of the relationship increases (becomes less negative) over time. This pattern is consistent with intensive growth during the Roman periods.

Figure 3 illustrates the relationship between building count and coin loss rate, by period. These results show, first, that there is no evidence for a relationship between population and coin loss for the Late Iron Age. Our sample is smallest for this period, but even accounting for the broad standard error on the slope of the relationship, it appears very unlikely that coins were used as a medium of exchange during this period. This is consistent with previous research, which has suggested that Iron Age coins were often used for religious offerings and gift exchange between rulers and subjects within local kingdoms (*72*, *106*, *107*). Second, across the Roman periods, the slope of the relationship is significantly greater than 1, and the intercept of the relationship increases (becomes less negative) over time. Table 1A reinforces these patterns by summarizing the estimated parameters of the scaling relationship for each period. These results show that the estimated slope for the three Roman periods excludes one and is within about one standard error of 7/6, the predicted value. This result indicates that, on average, coins were lost more rapidly in more populous settlements, with an elasticity consistent with the prediction of SST and similar to that observed for a wide range of socioeconomic rates in contemporary societies (*29*). Table 1 also shows a substantial increase in the intercept of the scaling relationship during the Roman era. This increase is statistically significant and suggest an increasing baseline rate of coin loss per capita, which in turn is consistent with an increasing number of coins circulating through households.

**Table 1.** Scaling analysis results. Notes: In all cases, the independent variable is the building count, and the dependent variable is listed above each register. Data were log-transformed prior to analysis. SE, standard error.

Group	Sample size	Intercept	SE	Slope	SE	F-Statistic	*P*
A. Coins loss per year, by period							
Late Iron Age	11	−4.625	0.420	0.070	0.445	0.158	0.8780
Early Roman*	120	−5.703	0.330	1.421	0.225	6.326	0.0000
Middle Roman	486	−4.945	0.156	1.233	0.085	14.531	0.0000
Late Roman*	133	−4.682	0.303	1.421	0.192	7.394	0.0000
B. Fine ware consumption rate, by period							
Late Iron Age	15	−3.658	1.282	1.273	0.781	1.629	0.1272
Early Roman^†^	67	−3.959	0.496	1.178	0.318	3.706	0.0004
Middle Roman	196	−3.322	0.182	1.270	0.086	14.815	0.0000
Late Roman^†^	46	−2.775	0.338	1.156	0.186	6.217	0.0000
C. Housing consumption, by period							
Late Iron Age^‡^	122	3.883	0.114	1.070	0.068	15.636	0.0000
Early Roman	131	3.981	0.122	1.093	0.078	13.954	0.0000
Middle Roman	146	4.064	0.124	1.183	0.097	12.165	0.0000
Late Roman^‡^	129	4.266	0.183	1.172	0.109	10.708	0.0000

*These differences are statistically significant (one-sided *t* = − 2.2805, df = 246.42, *P* = 0.01172).

†These differences are statistically significant (one-sided *t* = − 1.9736, df = 107.5, *P* = 0.02549).

‡These differences are statistically significant (one-sided *t* = − 1.7749, df = 212.45, *P* = 0.03867).

It is important to ask if these results might be a by-product of biased spatial sampling since previous studies have suggested that development in Roman Britain was more pronounced in the south and east. These areas are also the most developed today, and might be expected to lead to spatial biases in archaeological documentation as well. It is also important to assess the degree to which changes in the monetary system, especially inflation, might have degraded the purchasing power of an individual coin, thus undermining our interpretation. In the Supplementary Materials, we show that there is no association between period and region in the data, that the expected scaling of coin loss with population occurs even when the analysis is conducted by region, and that previous studies of Roman coinage have concluded that base token coins roughly maintained their purchasing power, despite debasement of their precious metal content and inflation in denominational prices over time. As a result, these concerns do not undermine our interpretation of patterns in the coin loss data as evidence of intensive economic growth.

### Fine ware consumption

**Fig. 4.**
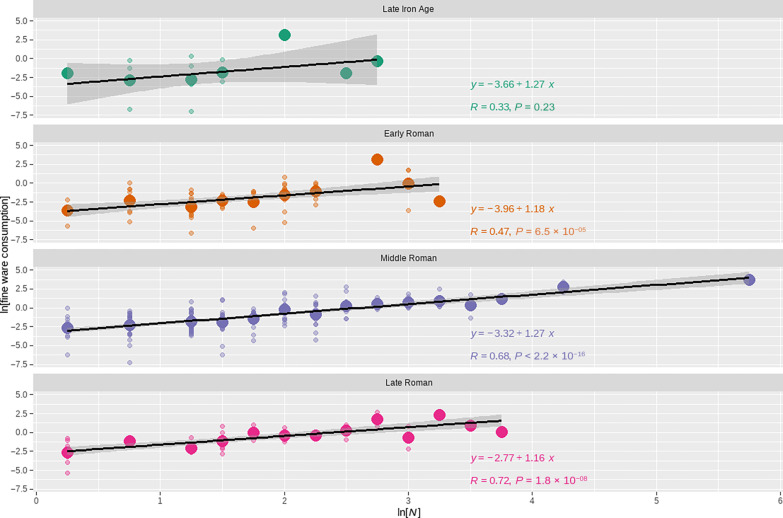
Relationship between fine ware consumption and population over time. The data have been binned by building count, in 0.25 increments. Small open circles represent individual cases, and larger filled circles represent the mean value for each bin. The relationship between coin loss rate and population is not statistically significant for the Late Iron Age, the slope of the relationship is relatively consistent and greater than 1 across the Roman periods, and the height of the relationship increases (becomes less negative) over time. This pattern is consistent with intensive growth during the Roman periods.

Patterns in consumption rates of fine ware pottery reinforce this interpretation of the coin loss data. Figure 4 illustrates the relationship between building count and fine ware consumption, by period, and Table 1B presents the analysis results. These show, first, that the scaling relationship has a consistent slope, even for the Late Iron Age, although the Late Iron Age sample size is too small for the observed relationship to exhibit statistical significance. Also, once again, across the Roman periods, the slope of the relationship is significantly greater than 1 and within one standard error of 7/6, the predicted value. In addition, the intercept of the relationship increases (becomes less negative) over time. This result indicates that, on average, fine wares were consumed more rapidly in more populous settlements, with the level of increasing returns to scale being consistent with the prediction of SST and similar to that observed for a wide range of socioeconomic rates (*29*). Also, as with coin loss, the increase in the intercept is statistically significant and suggests an increasing baseline rate of fine ware consumption over the course of the Roman period. This is consistent with increasing household inventories of fine wares and suggests broader increases in household possessions and the overall living standards as well.

In the Supplementary Materials, we consider if increasing substitution of metal plate for pottery could have led to the observed pattern. We conclude that any such substitution would be expected to reduce the slope of the scaling relation below that expected for a socioeconomic rate. Because this is not observed, it is not a viable alternative explanation. We also consider if impacts of colonial administration could have produced these patterns, finding that any such effects would be expected to show up in the residuals of individual settlements to the scaling relation rather than being reflected in the scaling relations themselves. As a result, these concerns do not undermine our interpretation of patterns in fine ware consumption as evidence of intensive economic growth.

### Housing consumption

**Fig. 5.**
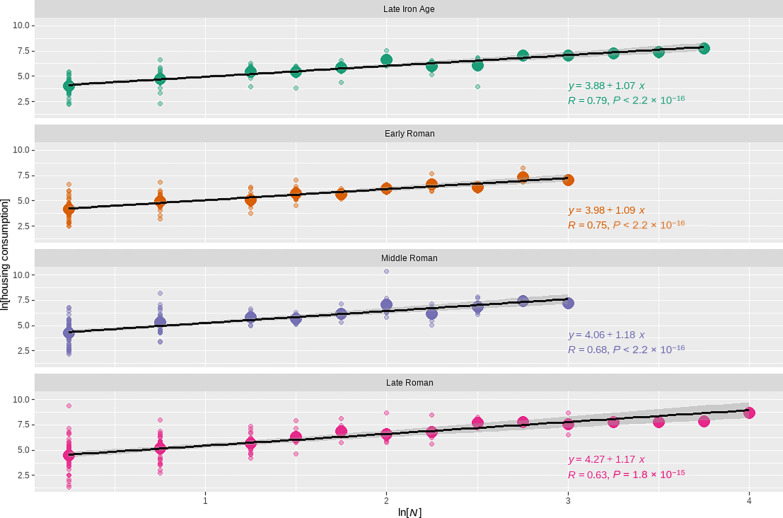
Relationship between housing consumption and population over time. The data have been binned by building count, in 0.25 increments. Small open circles represent individual cases, and larger filled circles represent the mean value for each bin. The slope of the relationship is relatively consistent and greater than 1, and the height of the relationship increases over time. This pattern is consistent with intensive growth.

The building area data are of a very different character than the coin and pottery data, in that the construction and demolition of buildings can often be dated individually, chronological sequences are often apparent from stratigraphic relationships, and the measure of housing consumption, the fraction of an excavated area taken up by residential buildings, derives from areas rather than counts of objects. Nevertheless, the building area data display the same scaling patterns observed for the other two measures. Figure 5 illustrates the relationship between building count and total building area, by period, and Table 1C presents the analysis results. These show, first, that the slope of the scaling relationship is consistently greater than 1 and is within one standard error of 7/6, the theoretical prediction, for the Middle and Late Roman periods. In addition, the intercept of the relationship increases over time. This result indicates that, on average, houses were larger and more densely packed in more populous settlements, with the level of increasing returns to scale being similar to that observed for a wide range of socioeconomic rates (*29*). As with coin loss and fine pottery consumption, the increase in the intercept is statistically significant and suggests an increasing baseline rate of housing consumption over the course of the Roman period. This is consistent with increasing household inventories and suggests broader increases in household possessions and the overall living standards as well.

## ACCOUNTING FOR INTENSIVE GROWTH

**Fig. 6.**
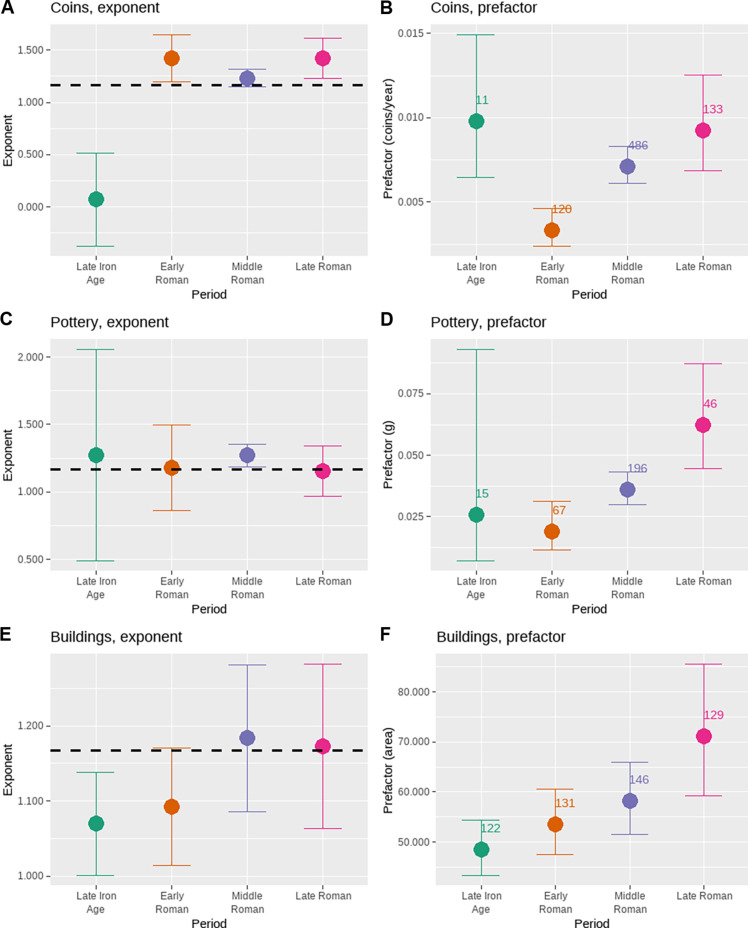
Relationships between population and baseline socio-economic rates over time. Panels show exponents and prefactors, by time period, for the relationship between building counts and coin loss per year [(**A**) and (**B**)], fine pottery consumption per year [(**C**) and (**D**)], and housing consumption [(**E**) and (**F**)], respectively. Data were transformed to logarithms prior to analysis; error bars represent standard errors using the White correction for heteroskedasticity; the dashed lines represent the expected exponent in SST; and numbers associated with the estimates in (B), (D), and (F) are the number of settlements included in each group. Note that, during the Roman periods, exponent estimates are greater than 1 and overlap with the SST expectation, and there is a consistent increase in prefactors.

The results presented above provide three independent lines of evidence for intensive (per capita) economic growth that was independent of agglomeration effects in Roman Britain over time. Figure 6 summarizes these results by presenting the estimated exponents and prefactors (the inverse log of the intercept) for each measure and period. It is remarkable that three different measures of socioeconomic activity, constructed in different ways and using different forms of evidence, should show such consistent scaling patterns. Eight of the 12 estimated exponents are within one standard error of the theoretical prediction of SST, and the 95% confidence interval for all but the Late Iron Age coin loss rate overlaps with this prediction. It is also noteworthy that the expected value of a proportional relationship, a slope of 1, lies outside the standard error of 8 of the 12 estimated exponents. These results provide strong evidence for increasing returns to scale, with a common elasticity, for a variety of socioeconomic rates in Romano-British society over a four-century period. In addition, the prefactors of the scaling relationships for all three measures show a consistent pattern of increase across Roman periods. These prefactors represent the number of coins lost per household-year, the grams of fine pottery broken per household-year, and the square meters of residential building in use per household-year, in a settlement consisting of a single household dating to a particular period. That all three baseline rates increased over time provides strong evidence that the overall economy of Britannia experienced intensive economic growth (increases in income per capita) over the four centuries that it was a Roman imperial province. We do not mean to suggest that there were not also disadvantages to indigenous Britons being subject to Roman rule, as several recent studies have pointed out (*70*, *87*, *108*, *109*). However, the overall long-term consequences of incorporation into the Roman Empire for the material conditions of life for the representative household seem undeniable.

Table 2 provides additional calculations that convert the intercepts of scaling relations from Table 1 into summaries of intensive economic growth. These calculations suggest that the average rate of intensive growth was on the order of a fraction of a percent per year across roughly century-long periods. The average rate of intensive growth, across all three measures, is 0.48% from the Early to Middle Roman period, and 0.28% from the Middle to Late Roman period. Table 2 also shows that, based on the change in prefactors over time, the baseline economic condition of households improved by a factor of about 2.5 between the Early and Late Roman periods. Of course, it is likely that growth rates varied substantially over shorter intervals and may well have been negative at times. In addition, it is important to ask if these three proxies reasonably capture what economists measure as gross domestic product (GDP), the total value of goods and services produced or consumed (exchanged) in an economy. Our measures encompass several sectors of the economy, including monetary incomes, consumption of household goods, and housing consumption, and it is also important that, in the Roman world, coins, pots, and houses were all produced by people other than the people who consumed them. These considerations suggest that the measures examined here do reasonably capture the overall economy. However, as previously mentioned, it is not possible to estimate the extensive growth rate (or the overall economic growth rate) using Eq. 9 because sufficient data to estimate changes in the center of the settlement system (see Fig. 1) are not available. Thus, the results presented here only inform on changes in per capita income deriving from institutional change and technological progress; they do not inform on additional growth related to demographic/agglomeration effects that may also have occurred. As such, our results likely underestimate the overall GDP growth. Nevertheless, they are important because they provide evidence for intensive growth in a preindustrial society.

**Table 2.** Calculation of average intensive growth rates across periods.

Period	Early Roman (50 to 150 CE)	Middle Roman (150 to 250 CE)	Late Roman (250 to 400 CE)
Midpoint	100	200	325
Years between midpoints	100	100	125
Intercept (coin loss)*	−5.703	−4.945	−4.682
Intercept (fine ware)*	−3.959	−3.322	−2.775
Intercept (buildings)*	3.981	4.064	4.266
Prefactor (coins/year per m^2^)^†^	0.003	0.007	0.009
Prefactor [fine/coarse (g)]^†^	0.019	0.036	0.062
Prefactor [building area (m^2^)]^†^	53.574	58.226	71.211
Prefactor ratio (coins)^‡^	1.000	2.136	2.777
Prefactor ratio (fine ware)^‡^	1.000	1.891	3.269
Prefactor ratio (buildings)^‡^	1.000	1.087	1.329
Mean of the three prefactor ratios^‡^	1.000	1.705	2.458
Growth rate (coin loss)^§^		0.76%	0.21%
Growth rate (fine ware consumption)^§^		0.64%	0.44%
Growth rate (housing consumption)^§^		0.08%	0.16%
Mean of the three growth rates^§^		0.49%	0.27%

*From Table 1.

†Calculated by taking the inverse logarithm of the corresponding intercept.

‡Calculated from the Early Roman baseline.

§Calculated from the previous period.

An obvious follow-up question raised by these results is whether it is possible to account for the factors that drove the observed rates of intensive growth. We return to the settlement scaling framework to provide some initial answers to this question. In the discussion of Eq. 4, above, it was noted that the intercepts of scaling relationships for socioeconomic rates are given by

$$Y_{0}\left(t\right)={G_{t}}^{2/3}/{\mu_{t}}^{1/3}$$ (10)

where *G_t_* represents the strength and energetic benefit of social interchange, and μ*_t_* is the energetic cost of movement. Thus, if *Y*_0_(*t*) increased over time, this implies that transport costs decreased, the energetic productivity of interaction increased, or some combination of the two. The role of reduced transportation costs, which facilitates interactions and exchange (of goods and information) and thus stimulates economic growth, has long been acknowledged (*110*). In a recent study, Wiseman *et al.* (*89*, *111*) estimated changes in transport costs in Roman Britain using distance-decay patterns of various pottery industries from their production locations. These estimates of changing transport costs allow one to isolate changes in the energetic consequences of social interchange that likely occurred. This analysis is presented in Table 3. We first average the three intercepts by period and then we convert this average to a prefactor and combine it with estimates of transport costs to estimate the value of interaction productivity, *G_t_*, based on Eq. 10. Last, because the estimates for *Y*_0_(*t*) and μ*_t_* are not in the same units, we convert the series of prefactors, transport costs, and interaction benefits to ratios to capture the relative change in each series. The results suggest that baseline productivity increased by about 2/3 between the Early and Middle Roman periods and increased again by the same factor between the Middle and Late Roman periods and that transport costs were reduced by a factor of about 2 between the Early and Middle Roman periods, with an additional slight reduction between the Middle and Late Roman periods. These, in turn, imply that the strength and productivity of social interchange increased by roughly a factor of 2/3 between the Early and Middle Roman periods and nearly doubled between the Middle and Late Roman periods. Thus, increases in the baseline productivity of individuals over time appear to have been driven by improvements in both μ*_t_* and *G_t_* between the Early and Middle Roman periods and by additional improvements in the productivity of social interchange *G_t_* between the Middle and Late Roman periods.

**Table 3.** Analysis of factors contributing to the observed intensive growth. Note: Values of μ*_t_* are from (*89*, *111*).

Period	Early Roman (40 to 150 CE)	Middle Roman (150 to 250 CE)	Late Roman (250 to 400 CE)
Intercept (coins)	−5.696	−4.955	−4.615
Intercept (fine ware)	−3.959	−3.273	−2.771
Intercept (housing)	3.981	4.064	4.266
〈ln *Y*_0_(*t*)〉	−1.891	−1.388	−1.040
*Y*_0_(*t*) (prefactor)	0.151	0.250	0.353
μ*_t_*	0.036	0.021	0.021
${\left[Y_{0}\left(t\right)\times {\mu_{t}}^{1/3}\right]}^{3/2}$	0.0111	0.0182	0.0301
*Y*_0_(*t*) ratio	1	1.655	2.343
μ*_t_* ratio	1	0.591	0.566
*G_t_* ratio	1	1.637	2.698

We can think of several factors that might have contributed to increases in *G_t_*. First, transaction costs may have declined through increasing Romanization (*72*), the gradual adoption of Roman identity that facilitated trust between anonymous strangers; the acceptance of Roman law, enforced by the judiciary and, ultimately, the military; and the increasing adoption of standardized Roman coinage to facilitate economic transactions (*112*, *113*). The fact that the layouts Romano-British towns tend to focus on visual and physical access to the forum-basilica complex at the central crossroads as opposed to religious temple complexes reinforces the importance of juridical institutions in promoting economic development. Second, changes in agricultural and food preparation technologies, including the use of more powerful draft animals, intensified meat production, increasing access to olive oil and wine, and increasing use of corn dryers and grain mills, likely increased the energy density of the effective environment (*74*). This would have translated into individuals being able to transfer more energy per exchange, overall and on average (*8*). While additional factors may also have been involved, we suspect that these broad sets of institutional and technological factors were most important for driving the observed increases in baseline productivity over time, with decreases in the energetic cost of movement playing a lesser role. We believe that additional research that seeks to quantify the effects of these specific institutional and technological factors would be fruitful.

## DISCUSSION: TOWARD A GENERAL FRAMEWORK

We have presented a formal and analytical framework for distinguishing extensive, agglomeration-driven, economic growth from intensive, system-wide institution-, and technology-driven growth. We have applied this framework in an analysis of proxy measures for population and socioeconomic rates derived from records of archaeological excavations in settlements dating from the Roman period in Britain. The results indicate that (i) there were consistent increasing returns to population scale for socioeconomic rates, with elasticities comparable to those observed for socioeconomic rates in cross-sectional data from contemporary urban systems and ancient agrarian societies; (ii) there was a consistent, modest increase in the baseline productivity of the settlement system over the course of the Roman period, at long-term average rates of a fraction of a percent per year, again consistent with time series data from contemporary urban systems, but in contrast to other past agrarian societies that have been examined to date; and (iii) this pattern of intensive growth appears to be attributable primarily to (institutional and technological) factors that increased the net energetic benefit of socioeconomic interchange.

These results are important in three ways. First, they illustrate the applicability and usefulness of SST for the study of economic change, both in the past and in the present. The fact that archaeological proxy data from a Roman Imperial province and direct observational data from contemporary nation-states can both be incorporated into a single analytical and theoretical framework that yields results that are consistent with a single set of expectations is notable and suggests that SST, as developed in the context of contemporary urban systems and as expanded to encompass past settlement systems of all scales, represents a positive step toward a general framework for investigating the evolution of human societies as complex systems (*4*, *17*, *19*, *28*, *42*).

Second, the results presented here provide evidence of intensive economic growth for a nonindustrial society, and they demonstrate that preindustrial economies could generate sustained intensive growth under specific conditions. The fact that intensive growth is apparent in fine ware and housing consumption is especially important because these measures are identical to those used in previous studies that found consistency in baseline rates over comparable spans of time in other preindustrial societies (*17*, *32*, *101*). The fact that these same measures reveal intensive growth in this case suggests that the absence of evidence for such growth in other preindustrial systems is evidence of absence. In addition, the fact that the same pattern is apparent across all three measures increases our confidence in the intensive growth interpretation.

We suggest that nonindustrial and contemporary economies may be differentiated more by the drivers and pace of economic growth than by the presence of intensive growth itself. Two recent works (*114*, *115*) follow a long tradition in arguing that the phenomenon of intensive economic growth is a defining feature of the present versus the past. This argument is not just that growth rates have been distinctly higher in the “modern era” but that the conditions for intensive growth themselves constitute “modernity.” We argue, in contrast, that, in certain ancient societies, institutional and technological change resulted in similar economic patterns of growth observed for the recent past. We are not implying a continuity between past and present, but rather allowing for breaks and changes in direction. Although there are some parallels between Roman Britain and the modern world, it is important to keep in mind that the economic history of Britannia need not have been characteristic of the Empire overall. Because of the way Britain was incorporated into an expanding institutional and technological frontier, intensive economic growth may well have been faster (and more easily measured archaeologically) in Britannia than elsewhere. The economic history examined here may thus be more relevant for processes of international development than for endogenously generated growth. Last, it is important to emphasize that, once the Roman legions left Britain in 410 CE, the economic system collapsed, and it did not recover to Roman levels for perhaps a millennium or more afterward (*116*).

Many past societies generated extensive economic growth, which increased per capita material outputs and living standards through agglomeration effects (*16*, *17*, *31*, *32*). Although the data examined here are only sufficient to identify intensive growth, it is likely that extensive growth also took place as the population of Britain increased and agglomerated into cities and other settlements following the Roman conquest. Thus, the identification of a preindustrial society in which both intensive and extensive economic growth occurred is important because it suggests that the differences between the economic systems of preindustrial and contemporary societies are more a matter of degree than kind. If so, additional studies of the economic history of past societies using SST would seem to offer great promise for a deeper understanding of the fundamental processes of socioeconomic development. We also wish to note that the signature of intensive growth is not the manner in which TFP grows—a topic of empirical dispute even for modern economies and the recent past (*117*)—but whether TFP can be identified and whether it grew.

Last, these results illustrate the potential for systematic synthesis of archaeological evidence from the contemporary cultural heritage management industry. The scale of documentation of the archaeological record has grown exponentially in recent decades, to the point that it is now reasonable to treat the accumulated quantified evidence as a type of sample-based “census” data, despite its partial and noisy nature and its coarse chronological resolution (*118*). There are increasing efforts to synthesize the cumulative archaeological record, in Britain and beyond, but many such efforts have proceeded in the absence of a strong problem orientation, as though the answers will become self-evident once enough data are collected. This study illustrates that useful knowledge does not emerge from evidence by itself but instead requires connecting this evidence to specific ideas and models that make concrete predictions that can be checked.

## Supplementary Materials

### This PDF file includes:

Supplemental Text Figs. S1 to S3 Tables S1 to S4 References
