# Supplementary material for: Identification and measurement of intensive economic growth in a Roman imperial province
Source: Sci Adv. 2024 Jul 5;10(27):eadk5517. doi: 10.1126/sciadv.adk5517 (PMC11225780; doi:10.1126/sciadv.adk5517)
Supplement: Supplementary file 1 — Supplemental Text Figs. S1 to S3 Tables S1 to S4 References [file sciadv.adk5517_sm.pdf]

Science Advances
AAAS

Supplementary Materials for

Identification and measurement of intensive economic growth in a Roman imperial province

Scott G. Ortman *et al.*

Corresponding author: Scott G. Ortman, scott.ortman@colorado.edu

Sci. Adv. **10**, eadk5517 (2024)
DOI: 10.1126/sciadv.adk5517

This PDF file includes:

Supplemental Text
Figs. S1 to S3
Tables S1 to S4
References

Provenance

The data utilized in this study derive from archaeological excavations at Romano-British settlements in England and Wales. Our primary sources are two large datasets prepared in the last decade: The Rural Settlement of Roman Britain (*119*) and the Defended Small Towns of Roman Britain (*120*). Raw data files for both datasets are available from the Archaeology Data Service (https://www.archaeologydataservice.ac.uk/). Both were compiled by research teams using compatible methods for categorizing and reporting data, which allowed us to combine their results in a single database. As the project names indicate, these two datasets cover rural settlements and small towns, but exclude primary towns. For the latter, we sourced comparable data from excavations in nine primary towns to the combined dataset. Table S1 summarizes the sources we consulted to obtain the data for primary towns. All settlements represent locations of multi-year residence. 'Primary towns' were administrative centers or *civitas* capitals and were the largest settlements in the province. Most were established shortly after the Roman Conquest, and many were built on former tribal centres or *oppida*. 'Small towns' did not have the administrative functions of primary towns and were generally smaller. They developed from the second century AD onwards, mostly along roads, although some originated as Roman military bases. 'Rural settlements' range from small farmsteads to large villas: what they all share is a focus on agricultural production, unlike the towns which also had administrative, manufacturing, and industrial roles.

Data Compilation Notes

In any large data compilation, there will be errors in the source data and errors made by the initial compilers. For example, in some cases tabulations of the coins from a site will combine site

finds and hoards but the compiler did not note as such in the original database. In other cases, the compiler may have simply entered the wrong number. These obvious data entry errors can often be identified as extreme outliers in visual summaries of the draft data. So, as our analysis proceeded, we double-checked extreme outliers by going back to the source reports, and we corrected data entry errors when encountered. No effort was made to document all edits we made relative to the archived versions of the Rural Roman Settlement Project data, but interested researchers can determine this by comparing our database to the archived data files. We estimate that such edits were made in a few dozen records (out of several thousand total records). We did not reinterpret the data or change the interpretations of the original excavators. We simply fixed errors in the original data compilations that formed the starting point for our database.

Assignment of materials to time periods

In this section, we first discuss the logic behind our relatively simple methods for assigning archaeological materials to time periods. It is useful to consider coin loss and fine ware pottery consumption separately from building consumption. If our goal were to assess coin loss and pottery consumption independent of population, our analysis could have proceeded by apportioning each line of evidence to time periods independently, based on the chronological significance of each object (see (*89, 111*) for an example). However, here we are interested in relationships between coin loss, pottery consumption, and the associated population, reflected in the residential building count, and any method that apportioned these materials independent of building counts would destroy such relationships. Because our goal is to estimate accumulation rates of materials relative to the number of people who lived in an area, it is not feasible to associate coins and pots with the occupations of specific buildings based on their contexts because most excavation contexts contain mixed materials from multiple phases of occupation in that area. It is also not feasible to apportion

coins and pottery by dating individual objects themselves. For example, using coin issue dates to associate coins with buildings dated to different phases imposes the assumption of a constant relationship between coin age and coin loss, when aggregate issue date distributions show this assumption is not reasonable (*84*). Also, using the production spans of different pottery types to associate pottery with dated buildings is not reasonable because most coarse wares are datable only to the Roman period overall. As a result, this approach one forces one to apportion most pottery evenly across phases, or proportionately to the dated buildings. This would impose accumulation rates on the material when our goal is to measure these rates relative to the associated population. Due to these sorts of factors, for the questions we are asking there is no alternative but to consider all coins and pottery from an excavation as having accumulated over the inclusive occupation span of all associated buildings. Our solution for coins and pottery was thus to capture the total number of coins, total weight of fine and coarse pottery, the total number of buildings encountered within an excavation area, and the beginning and end date of the overall occupation. Then, we assigned these summary measures to a period based on the midpoint of the occupation dates. We essentially followed the methodology used in paleobiology: to calculate rates of interest for a site in a specific location, and then arrange these sites in chronological order based on the midpoint of the date range (*121, 122*).

Many sites and/or buildings are associated with dates that span the entire Roman Period. Such cases may represent long occupations, or cases where the occupations are imprecisely dated. Out method treats these two situations the same way and generally assigns the associated materials to the Middle Roman Period (the midpoint between 50 and 400 AD is 225 AD) unless the excavators clearly indicated that the bulk of the material dated to the Early or Late period. Note also that a site or building dating between 150 and 400 AD will be assigned to the Late Roman

Period (midpoint = 275 AD) and a site or building dating between 50 and 250 AD will be assigned to the Early Roman Period (midpoint = 150 AD). Although the materials recovered from an excavation were rarely deposited *only* during the period to which they were assigned, more complex methods of apportioning coins and pottery to time periods are not appropriate, as discussed above.

The situation for building areas is much simpler than is the case for coins and pottery because, in this case, building areas are directly associated with building counts, and the construction and abandonment dates of buildings can be determined from the most recent coins and potsherds found immediately below the walls and floors or immediately above them, respectively. So, for each excavation, we assigned each building to one of our four periods based on the information provided in the excavation reports, and then we counted the number of buildings dating to each period and calculated the total area of these buildings that was exposed within the excavation area. When a building was only partly exposed, we included only that area in the calculation. We did not extrapolate beyond the excavation area. The issues with time averaging discussed above with respect to coins and pottery are less relevant for buildings.

The excavated area represented by each "site" varies substantially, as does the number of excavations associated with each settlement. Most small Roman settlements are known only from a single excavation, but larger settlements—particularly those beneath still-occupied towns—have often been exposed in several different excavations. To account for these situations, we aggregated the measures we calculated for each excavation by settlement and period, as discussed in the main text. For some primary towns we were also able to assign the excavated materials to different periods. As examples: at Exeter a recent synthesis assigned deposits to the era of the initial fortress and an early vs. later civil period (*123, 124*); at Colchester, deposits were assigned to the initial

fortress, the town prior to its destruction during the Boudiccan revolt ca. 60 CE, and the post-Boudiccan town (assigned here to the Middle Roman period) (*125*); and at Verulamium, the excavators distinguished pre-Boudiccan deposits from a layer of timber buildings that were burned approximately 160 CE, and an uppermost layer of Late Roman stone buildings (*126*). Tables S2 and S3 summarizes the resulting dataset, by settlement type and region, respectively.

Table S2 shows that for all three measures there are statistical associations between period and settlement size in the dataset. This is due to two factors. First, our method of assigning excavations to periods places sites occupied across the Roman period, sites imprecisely dated to the overall Roman period, and sites occupied only during the middle years of the Roman period, into the same chronological group. Second, Romano-British society was largely rural, and areas chosen for excavation relate to present-day development. As a result, excavated areas represent an approximate simple random sample of all settlements, most of which are small. In contrast, Table S3 shows that for all three measures there is no statistical association between period and region of the Roman province. This is important because studies have shown that Romanization and economic development were more pronounced in the south and east than in the west and northwest (*71, 73*). This lack of association between region and period is important because it implies changes in the prefactors of scaling relations over time are not an artifact of spatial sampling. However, this does not rule out the possibility that the slopes of scaling relations are a byproduct of sampling across regions with markedly different levels of economic development. This possibility is further evaluated below.

Interpretation of measures as socio-economic rates

As with any compilation generated by multiple investigators, one would expect the underlying data to contain substantial variation in methodology and accuracy. For this reason, one should not put too much emphasis on the observed values for individual excavation sites and settlements. Fortunately, these shortcomings are not insurmountable. It is reasonable to suggest that, at the scale of the entire database, the many sources of error in individual measurements tend to cancel each other out, such that summary statistics for groups of sites are reasonably accurate and unbiased. In addition, the measures we examine exhibit properties that are consistent with socio-economic measures. In contemporary societies, such measures generally exhibit lognormal distributions for which the mean increases with development. Figure S1 presents histograms of the three measurements considered in this study across all settlements assigned to the Roman period, and Table S4 presents summary statistics of the associated raw values. In all three cases, the data samples exhibit approximate lognormal distributions, and the within-period means show increases over time, as one would expect based on previous research on the Roman economy. Socio-economic rates also tend to be faster in larger settlements than in smaller ones. Although measured site areas are not consistently available, most excavations are assigned to a settlement size category, and this classification can be used to assess the relationship between our three measurements and settlement size in a rough way. Figure S2 summarizes these data and shows that means of the log-transformed measures vary as one would expect for socio-economic measures.

Finally, it is important to consider whether our measures of coin loss, fine pottery consumption, and housing consumption reflect the accumulated wealth of households instead of socio-economic rates related to incomes. For coins, we would expect hoards to provide a useful

measure of wealth accumulation if they could be associated with a population proxy, but because most site finds are low-value coins these are more likely to reflect monetary value that flowed through a household over time, a socio-economic rate related to the household's income. The pottery found in an excavation also does not represent a store of usable vessels, but an accumulation of broken potsherds. As such, the accumulation rates of pottery reflect a socioeconomic rate as well. Finally, although there is a tradition in archaeology that interprets residential building area as an index of household wealth (*99, 127*), most wealth accumulation takes multiple generations and involves the transformation of physical product into social obligations mediated through money or other forms of formal or informal accounting (*128*). In the Roman world most incomes were generated within or adjacent to residences, and these residences needed to be large enough to house the family, temporarily store household product, and store household possessions, most of which depreciated through use. Finally, because wealth reflects accumulated surplus, one would expect wealth accumulation to be faster in households with larger incomes, and this would lead wealth measures to exhibit steeper scaling coefficients than are characteristic of socioeconomic rates ($\beta > 7/6$). Since such extreme scaling is not observed, we argue residential building area is also better thought of as a measure of income (the space through which resources flow) than as a measure of wealth (an accumulated stock of largely nonphysical resources).

Alternative explanations for the observed scaling relationships

Here, we consider two alternative explanations for the coin loss data. First, recent studies of Roman Britain have argued that the degree to which local residents adopted Roman culture varied across the province, with Romanization generally being more apparent in the southern and

eastern portions of England than in the west, north, and Wales (*70, 71, 73*). It is therefore important to check whether the scaling relations identified above are a byproduct of spatial heterogeneity in the role of Roman coins for everyday transactions. Figure S3 summarizes the parameters of the relationship between buildings and coin loss rates by region, using subdivisions established by the Rural Roman Settlement project. These results show less consistent patterns than are observed for chronological groupings, but they do still show that coins generally exhibit the expected super-linear scaling across much of the province, with the possible exceptions of the West and Wales. They also show that baseline coin loss rates were indeed higher overall in eastern and southern England than in the central belt and north-east. These results reinforce the conclusion that levels of coin use varied across the province; but nevertheless, there is no association between period and region in this dataset (see Table S3) so the spatial pattern of coin use is not likely responsible for the evidence of increase in overall baseline coin loss rates over time.

Second, it is important to ask whether the coin loss results in the main text are a byproduct of changes in the monetary system and prices as opposed to increases in baseline productivity. Recent research on Roman coins in Britain has tended to view increases in the prevalence of coins over time as evidence of the gradual penetration of Roman monetary practices into British society (*106, 129*). While this is surely true, the other lines of non-monetary evidence reviewed in the main text suggest improving living standards did accompany this increase. The significance of this association depends on the purchasing power of the typical lost coin over time. Previous numismatic studies suggest Roman consumers generally viewed the purchasing power of their base silver coins, either denarii or nummi, as being tied to their precious metal content (*130*). Indeed, the initial concept of the coin was a standard weight of precious metal, guaranteed by the issuing political authority as indicated by the stamp. In addition, studies of price information from

Roman Egypt suggest consistency in exchange ratios by weight of wheat, silver, and gold; and increasing wages for several professions relative to these ratios over time (*38*, *131*).

The precious metal content of Roman base coins gradually decreased over time, especially during the 3rd century, leading to substantial inflation in denominational prices. Given this, the key question for present purposes is how prices evolved, not relative to the denominational values or precious metal content of the base coins, but relative to individual token coins representing small change that make up the bulk of coin loss assemblages. Previous studies of this question have concluded that Roman coinage evolved in such a way that token coins maintained their purchasing power even as their face values changed. For example, Harl (*130*) concludes that, until about 250 CE, a middle-sized bronze coin weighing between one-third and one-half of a Roman ounce passed as the coin of common transactions, easily buying a loaf of bread, with its heavier multiple being sufficient to purchase daily subsistence. The face value of such coins did not matter so much as their constant power for purchasing staples, and the ready exchange of these token coins into higher-value base coins. And as the precious metal content of silver base coins declined, prices rose in such a way that these base coins gradually became token coins, as occurred with the nummus, a silver washed bronze coin introduced in 293 CE. These conclusions suggest the net effect of changes in the Roman monetary system over time was a rough consistency in the purchasing power of the basic token coin, regardless of its face value. This would in turn imply that coin loss rates were proportional not only to the number of coins in circulation, but also the purchasing power of households. If so, the pattern shown in Figures 3 and 6 in the main text reflect consistently higher real incomes in larger settlements and increasing baseline real incomes across all households over time.

We also consider two possible alternatives regarding the fine ware consumption results. One possible alternative is that the observed pattern derives from an increasing substitution of metal plate for pottery over time. Several 4th century CE hoards of silver and pewter (lead alloyed with tin) tableware have been found, and authors have suggested that pewter in particular provided a lower cost substitute for silver plate in the 3rd and 4th centuries (*109*). In addition, fine ware pottery of several industries, including Samian ware and various color-coat wares, have molded decorations that mimic the appearance and production technique of silver tableware with chased (hammered from the reverse side) decoration, as exemplified by the Mildenhall Treasure. These various lines of evidence suggest that fine ware pottery was increasingly in competition with metal tableware, and that as real household incomes increased, fine ware pottery may have been increasingly replaced by pewter and silver tableware. Metal tableware is much less fragile and has a much longer use-life than fine ware pottery; but except for those rare objects that were buried for safekeeping and then forgotten, most metal tableware was eventually melted down and reused. As a result, it is not realistic to measure pewter or silver tableware consumption across settlements and over time, as is the case for pottery. However, if substitution of metal for pottery was a major factor, one might expect such replacement to have been more prevalent in larger settlements, where coin loss rates suggest household incomes were generally higher, and this would have the effect of reducing the slope of the scaling relationship between building count and fine ware consumption over time. Figures 4 and 6 in the main text suggest that this effect, if present at all, was very slight. In addition, widespread replacement of fine wares by metal plate would have reduced fine ware consumption rates overall, and thus reduced the intercept of the scaling relationships. This is opposite the observed pattern. So, even if there was some replacement of fine wares by metal over

time, it does not appear to have been substantial enough to obscure the pattern of intensive growth revealed by these data.

A second possible alternative derives from comparisons of the material culture of Early Roman cities and adjacent rural sites, which have concluded that cities founded after the Roman conquest functioned primarily as parasitic foci of government and administration, with limited economic impact or draw for existing local populations (*87, 108*). A primary line of evidence offered in support of this view is a higher rate of fine ware consumption in Early Roman contexts at Colchester and London than in surrounding smaller settlements. One might therefore ask whether patterns in fine ware consumption might reflect colonialism more than economic development. This seems unlikely, for three reasons. First, the theoretical expectation for the slope of scaling relations for socio-economic rates like fine pottery consumption derive from basic considerations of social and infrastructural networks embedded in space and time, so under this model one would generally expect fine ware consumption rates to be faster in larger settlements. Indeed, one can think of SST as a means of controlling for the effects of scale regarding socio-economic rates. So, if the parasitic model of early Romano-British cities is correct one would expect to see evidence of parasitism in the form of positive residuals from the predicted scaling, which are in effect scale-adjusted urban indicators (*132*). This would show up in the form of an upward skew in the residuals in the upper tail of scaling plots. This is not apparent in Figure 4 of the main text. Second, the slope of the scaling relationship between fine pottery consumption and population matches the theoretical expectation for a socio-economic rate for the entire period considered by this study, and similar scaling has been observed for a range of socio-economic measures for societies with a wide range of economic and sociopolitical structures (*16, 28, 32, 100, 101*). Given this, it is difficult to imagine that biases induced by colonial administration could

have produced the observed scaling relations. Finally, the consistent increase in the intercept of the scaling relation over time is consistent with economic development and inconsistent with a model in which fine ware consumption was primarily a byproduct of access to these products through colonial administrative networks over a four-century period. We do not mean to suggest that signatures of colonialism might not also be embedded in these data, but if they are, they would be reflected in residuals to the scaling relations we observe rather than the scaling relations themselves.

Data Availability

Upon publication, the data files utilized in this article will be made available at: https://core.tdar.org/project/392021/social-reactors-project-datasets.

Table S1. Sources consulted for data from excavations in primary towns.

**Site (Excavations)**	**References**
Caerwent (Time Team)	(*133*)
Cirencester (St. Michaels and Town Centre, Beeches Road)	(*134, 135*)
Colchester (Fortess, Boudiccan, Roman)	(*125, 136-138*)
Exeter (Cathedral Close, 1971-9)	(*123, 124, 139*)
Leicester (Causeway Lane)	(*140*)
Silchester (Defences, Insula IX, Mapping)	(*141-143*)
Verulamium (Frere)	(*126, 144, 145*)
Wroxeter (Fortress, Baths, Basilica, Insula X, Lining holes, Exc. Committee)	(*146-152*)
Dorchester (Colliton Park, Old Methodist Chapel)	(*153, 154*)

Table S2. Summary of the analysis data, by period, settlement type, and data type.

Period	Settlement Size<br>Small (<1-3 ha)	medium (4-8 ha)	Large (9+ ha)	small town	primary town	fortress	Not Specified	Total
Coins (*Chi*-square = 57.85, *df* = 18, *P* = 4.532e-06)								
Late Iron Age (pre-50 CE)	7	2	1				1	11
Early Roman (50-150 CE)	61	17	6	6	6	3	21	120
Middle Roman (150-250 CE)	195	123	54	13	7		94	486
Late Roman (250-400 CE)	34	38	13	12	6		30	133
Total	297	180	74	31	3	19	146	750
Pottery (*Chi*-square = 51.274, *df* = 18, *P* = 4.833e-05)								
Late Iron Age (pre-50 CE)	10	1					4	15
Early Roman (50-150 CE)	39	8	2	3	4	3	8	67
Middle Roman (150-250 CE)	97	30	17	9	6		37	196
Late Roman (250-400 CE)	8	14	5	7	5		7	46
Total	154	53	24	19	3	15	56	324
Building areas (Chi-square = 66.685, *df* = 15, *P* = 1.728e-08)								
Late Iron Age (pre-50 CE)	114	27	9	1			27	178
Early Roman (50-150 CE)	96	46	33	9	8		40	232
Middle Roman (150-250 CE)	104	89	31	9	8		45	286
Late Roman (250-400 CE)	93	83	34	13	7		58	288
Total	407	245	107	32	0	23	170	984

Notes: Numbers in cells indicate the number of settlements for which each data type is available. All settlements are also associated with building counts. Data for three fortresses derive from excavations within Exeter, Colchester, and Wroxeter. For all three measures there is an association between settlement size and period due to our method of assigning excavations to periods, and the fact that most Roman settlements were small.

Table S3. Summary of the analysis data, by period, region, and data type.

Period	Region						
	South	East	Central Belt	North-East	West and Wales	North	Total
Coins (*Chi*-square = 16.398, *df* = 15, *P* = .3226)							
Late Iron Age (pre-50 CE)	3	1	7	0	0	0	11
Early Roman (50-150 CE)	30	12	47	15	14	2	120
Middle Roman (150-250 CE)	129	49	226	32	38	12	486
Late Roman (250-400 CE)	28	8	73	11	12	1	133
Total	190	70	353	58	64	15	750
Pottery (*Chi*-square = 12.749, *df* = 12, *P* = .3875)							
Late Iron Age (pre-50 CE)	1	0	12	2	0	0	15
Early Roman (50-150 CE)	12	5	33	7	10	0	67
Middle Roman (150-250 CE)	34	16	121	11	14	0	196
Late Roman (250-400 CE)	9	4	27	2	4	0	46
Total	56	25	193	22	28	0	324
Building areas (*Chi*-square = 18.651, *df* = 15, *P* = 0.23)							
Late Iron Age (pre-50 CE)	38	11	64	28	29	8	178
Early Roman (50-150 CE)	54	21	89	26	27	15	232
Middle Roman (150-250 CE)	76	26	110	26	36	12	286
Late Roman (250-400 CE)	66	19	134	24	34	11	288
Total	234	77	397	104	126	46	984

Notes: Numbers in cells indicate the number of settlements for which each data type is available. All settlements are also associated with building counts. Note that there is no evidence for association between region and period for any of these measures.

Table S4. Summary statistics for the raw measures considered in this paper.

Period	Number of excavations	Min.	Max.	Median	Mean	SD
Mean building area per excavation (m^2)						
Late Iron Age	122	5.0	378.6	70.9	77.8	55.11
Early Roman	131	12.0	757.6	70.9	94.0	96.07
Middle Roman	146	9.0	4354.2	78.0	160.4	401.95
Late Roman	129	3.7	12000.0	95.9	273.3	1067.52
Coin loss per year of occupation and per ha of excavation						
Late Iron Age	124	0.0006	0.5846	0.00	0.07	0.17
Early Roman	260	0.0003	30.9524	0.08	1.66	4.64
Middle Roman	672	0.0001	166.6667	0.23	2.66	11.06
Late Roman	180	0.0007	302.5000	1.12	9.70	34.94
Ratio of fine ware to coarse ware pottery						
Late Iron Age	124	0.0009	22.4481	0.14	1.82	5.72
Early Roman	260	0.0013	22.7164	0.13	0.75	2.89
Middle Roman	672	0.0007	39.8370	0.28	1.32	3.99
Late Roman	180	0.0047	14.0380	0.37	1.45	2.87

Figure S1. Histograms of the measures considered in this paper by Roman period; A, mean building area per excavation; B, coins per year of occupation per hectare of excavation; C, ratio of fine ware to coarse ware. Note that all distributions are approximately log-normal, and that the mean-log value of all three measures increases over time.

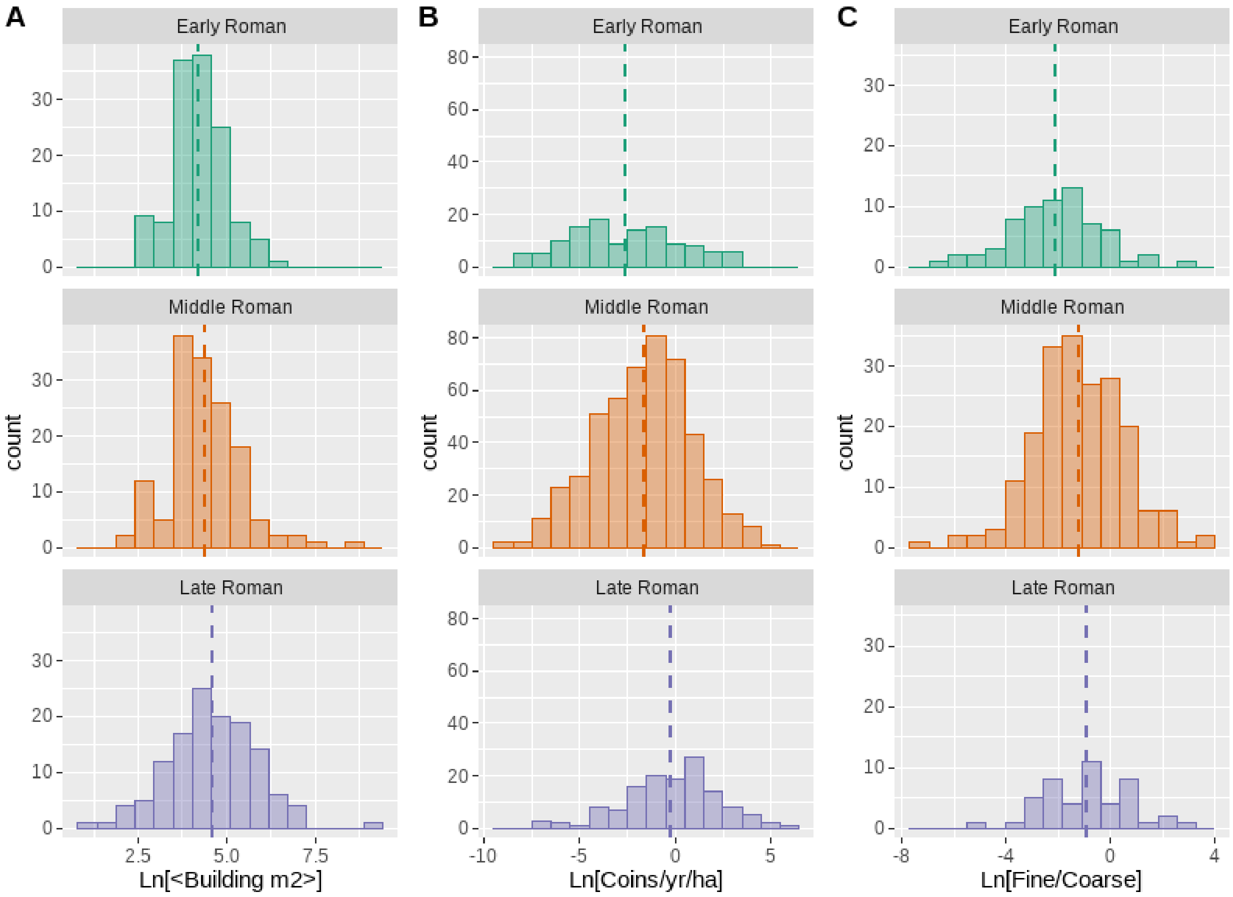

Figure S2. Variation in the three measures considered in this paper, by settlement size category. Filled circles represent group means, and error bars represent standard deviations. Note that the means of all three measures increase with settlement size category, with the notable exception of building areas in medium-sized sites. This reflects the inclusion of villas in that size category. Variation in these measures is consistent with their identification as socio-economic rates.

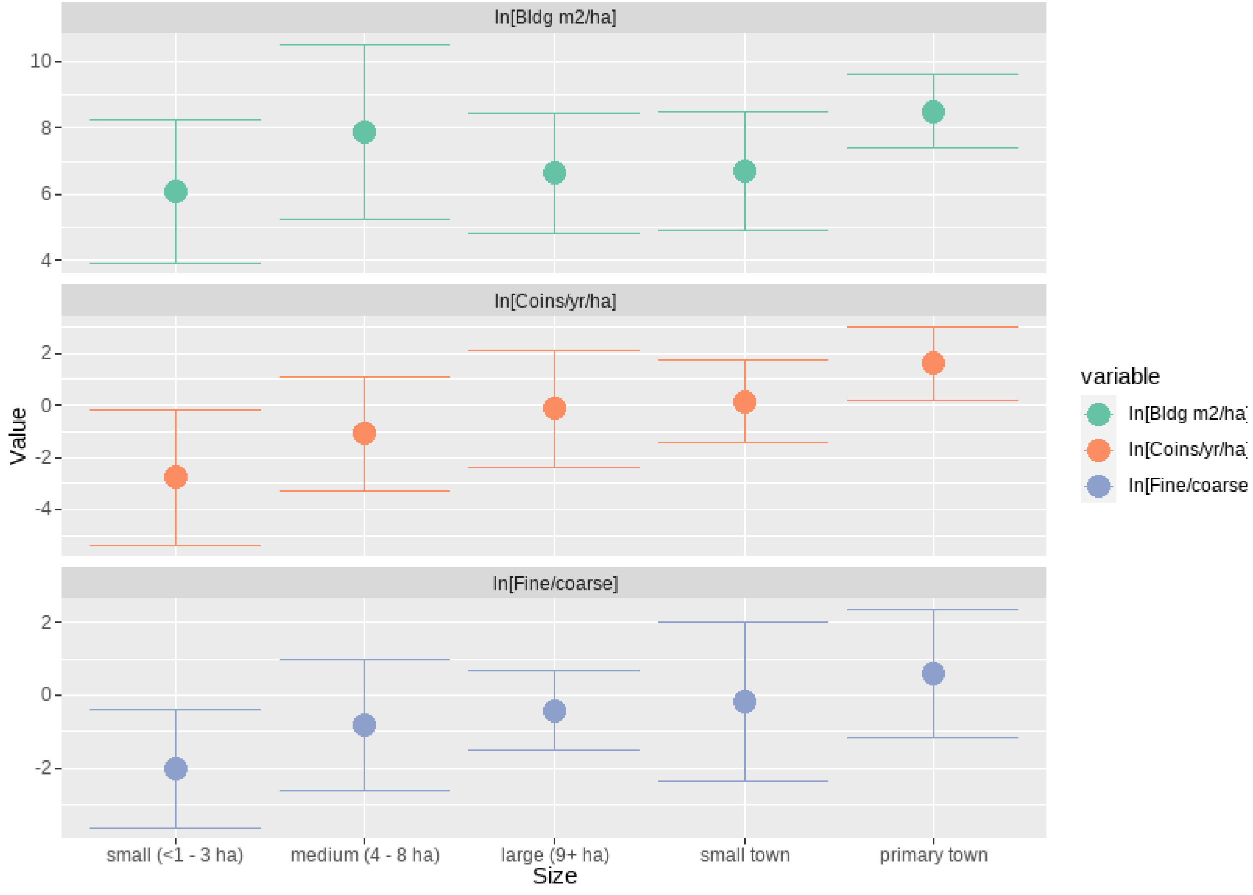

Figure S3. Slopes (A) and prefactors (B) for the relationship between building count and coin loss rate, by region. Data were log-transformed and binned by size prior to analysis; Late Iron Age settlements were excluded; error bars represent standard errors using the White correction for heteroskedasticity; the dashed line in (A) represents the expectation from Settlement Scaling Theory; and numbers associated with the estimates in (B) represent the number of settlements included in each group. Note the roughly consistent parameters for all regions but the West and Wales. This result provides evidence of spatial variation in the use of coins.

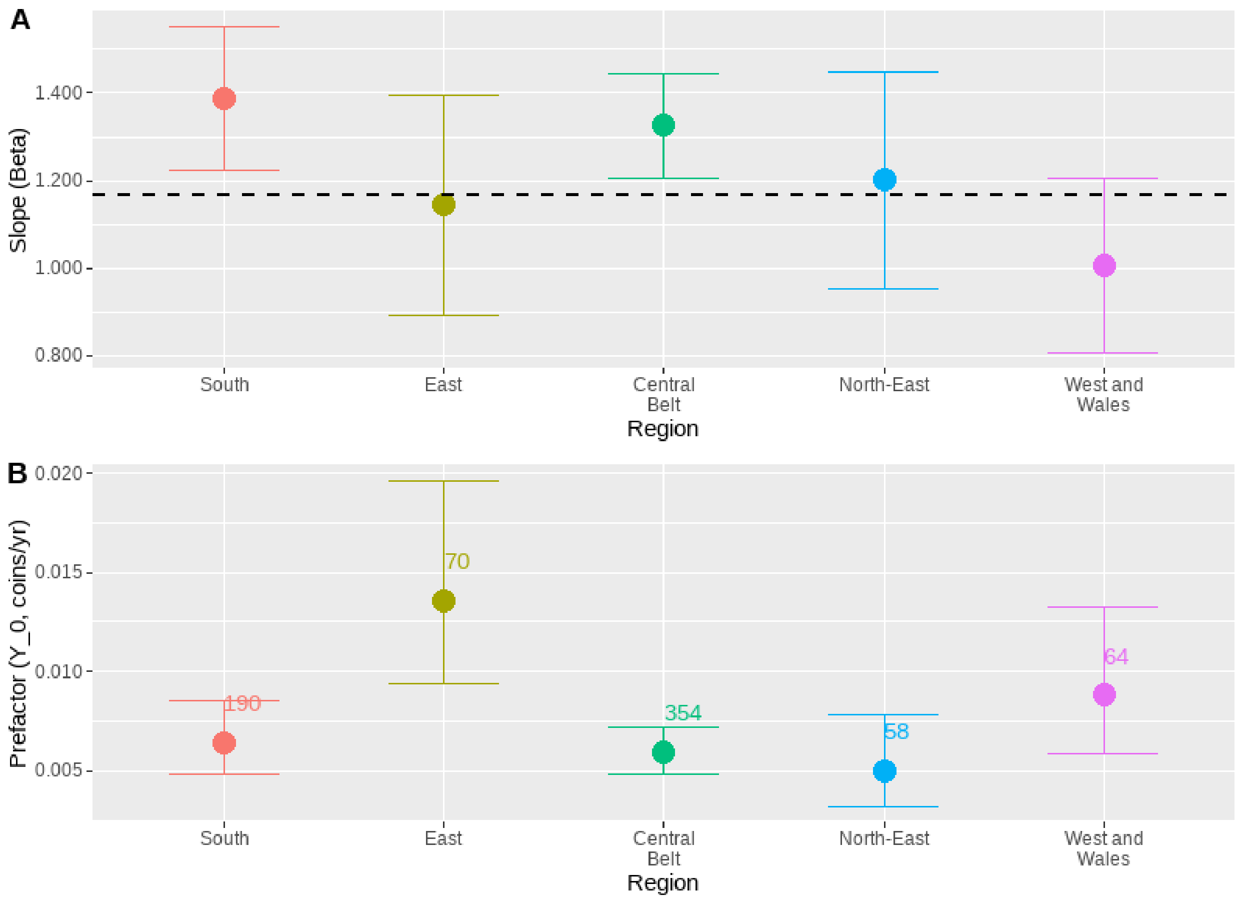

REFERENCES AND NOTES

1. C. I. Jones, P. M. Romer, The new Kaldor facts: Ideas, institutions, population, and human capital. *Am. Econ. J. Macroecon.* **2**, 224–245 (2010).

2. J. V. Henderson, Urbanization and economic development. *Ann. Econ. Financ.* **4**, 275–341 (2003).

3. E. L. Glaeser, J. D. Gottlieb, The wealth of cities: Agglomeration economies and spatial equilibrium in the United States. *J. Econ. Lit.* **47**, 983–1028 (2009).

4. J. Lobo, L. M. A. Bettencourt, M. E. Smith, S. Ortman, Settlement scaling theory: Bridging the study of ancient and contemporary urban systems. *Urban Stud.* **57**, 731–747 (2020).

5. R. J. Barro, X. Sala-i-Martin, *Economic Growth* (MIT Press, ed. 2, 2004).

6. C. I. Jones, *Introduction to Economic Growth* (W. W. Norton & Company, 2013).

7. J. Mokyr, in *Handbook of Economic Growth*, P. Aghion, S. Durlauf, Eds. (North Holland, 2006).

8. V. Smil, *Energy and Civilization: A History* (MIT Press, 2017).

9. E. A. Wrigley, *The Path to Sustained Growth: England's Transition from an Organic Economy to an Industrial Revolution* (Cambridge Univ. Press, 2016).

10. J. Mokyr, *A Culture of Growth: The Origins of the Modern Economy* (Princeton Univ. Press, 2018).

11. A. Young, The tyranny of numbers: Confronting the statistical realities of the East Asian economic miracle. *Q. J. Econ.* **110**, 641–680 (1994).

12. M. Fujita, P. Krugman, A. J. Venables, *The Spatial Economy: Cities, Regions, and International Trade* (MIT Press 1999).

13. K. J. Arrow, in *The Return to Increasing Returns*, J. M. Buchanan, Y. J. Yoon, Eds. (Univ. of Michigan Press, 1994), pp. 69–84.

14. M. Kelly, The dynamics of Smithian growth. *Q. J. Econ.* **112**, 939–964 (1997).

15. R. R. Nelson, H. Pack, The Asian miracle and modern growth theory. *Econ. J.* **109**, 416–436 (1999).

16. S. G. Ortman, Z. Cooper, Artifact density and population density in settlement pattern research. *J. Archaeol. Sci. Rep.* **39**, 103189 (2021).

17. S. G. Ortman, J. Lobo, Smithian growth in a nonindustrial society. *Sci. Adv.* **6**, eaba5694 (2020).

18. L. M. A. Bettencourt, *Introduction to Urban Science: Evidence and Theory for Cities as Complex Systems* (MIT Press, 2021).

19. L. M. A. Bettencourt, Urban growth and the emergent statistics of cities. *Sci. Adv.* **6**, eaat8812 (2020).

20. L. M. A. Bettencourt, Towards a statistical mechanics of cities. *C. R. Phys.* **20**, 308–318 (2019).

21. E. Boserup, *Population and Technological Change: A Study of Long-Term Trends* (Univ. of Chicago Press, 1981).

22. P. M. Romer, Endogenous technological change. *J. Polit. Econ.* **98**, S71-S102 (1990).

23. M. Kremer, Population growth and technological change: One million B.C. to 1990. *Q. J. Econ.* **108**, 681–716 (1993).

24. R. L. Carneiro, The transition from quantity to quality: A neglected causal mechanism in accounting for social evolution. *Proc. Natl. Acad. Sci. U.S.A.* **97**, 12926–12931 (2000).

25. C. I. Jones, in *Knowledge, Information, and Expectations in Modern Macroeconomics: In Honor of Edmund S. Phelps*, P. Aghion, F. Frydman, J. Stiglitz, M. Woodford, Eds. (Princeton Univ. Press, 2003), pp. 498–521.

26. M. Nag, Anthropology and population: Problems and perspectives. *Popul. Stud.* **27**, 59–68 (1973).

27. J. Simon, *Theory of Population and Economic Growth* (Basil Blackwell, 1986).

28. L. M. A. Bettencourt, The origins of scaling in cities. *Science* **340**, 1438–1441 (2013).

29. L. M. A. Bettencourt, J. Lobo, D. Helbing, C. Kühnert, G. B. West, Growth, innovation, scaling, and the pace of life in cities. *Proc. Natl. Acad. Sci. U.S.A.* **104**, 7301–7306 (2007).

30. X. Liu, Z. Zou, Analysis on urban scaling characteristics of China's relatively developed cities. *PLOS ONE* **15**, e0236593 (2020).

31. S. Klassen, S. G. Ortman, J. Lobo, D. Evans, Provisioning an early city: Spatial equilibrium in the agricultural economy at Angkor, Cambodia. *J. Archaeol. Method Theory* **29**, 763–794 (2021).

32. S. G. Ortman, A. H. F. Cabaniss, J. O. Sturm, L. M. A. Bettencourt, Settlement scaling and increasing returns in an ancient society. *Sci. Adv.* **1**, e1400066 (2015).

33. I. M. Morris, Economic growth in ancient Greece. *J. Inst. Theor. Econ.* **160**, 709–742 (2004).

34. P. Temin, *The Roman Market Economy* (Princeton Univ. Press, 2013).

35. J. Ober, *The Rise and Fall of Classical Greece* (Princeton Univ. Press, 2015).

36. J. G. Manning, *The Open Sea: The Economic Life of the Ancient Mediterranean World from the Iron Age to the Rise of Rome* (Princeton Univ. Press, 2018).

37. A. Bresson, *The Making of the Ancient Greek Economy: Institutions, Markets and Growth in the City States* (Princeton Univ. Press, 2015).

38. K. Harper, People, plagues, and prices in the Roman world: The evidence from Egypt. *J. Econ. Hist.* **76**, 803–839 (2016).

39. W. Scheidel, In search of Roman economic growth. *J. Roman Archaeol.* **22**, 46–70 (2009).

40. R. Saller, in *The Roman Economy*, W. Scheidel, Ed. (Princeton Univ. Press, 2012).

41. M. E. Smith, S. G. Ortman, J. Lobo, C. E. Ebert, A. E. Thompson, K. M. Prufer, R. Liendo Stuardo, R. M. Rosenswig, The low-density urban systems of the Classic period Maya and Izapa: Insights from settlement scaling theory. *Lat. Am. Antiq.* **32**, 120–137 (2021).

42. S. G. Ortman, M. E. Smith, J. Lobo, L. M. A. Bettencourt, Why archaeology is necessary for a theory of urbanization. *J. Urban Archaeol.* **1**, 151–167 (2020).

43. E. L. Glaeser, H. D. Kallal, J. A. Scheinkman, A. Shleifer, Growth in cities. *J. Polit. Econ.* **100**, 1126–1152 (1992).

44. E. L. Glaeser, J. A. Scheinkman, A. Shleifer, Economic growth in a cross-section of cities. *J. Monet. Econ.* **36**, 117–143 (1995).

45. R. Hausmann, C. A. Hidalgo, The network structure of economic output. *J. Econ. Growth* **16**, 309–342 (2011).

46. E. L. Glaeser, *The Economic Approach to Cities*, Harvard Institute of Economic Research Discussion Paper no. 2149 (Harvard Univ., 2008).

47. L. Alessandretti, P. Sapiezynski, V. Sekara, S. Lehmann, A. Baronchelli, Evidence for a conserved quantity in human mobility. *Nat. Hum. Behav.* **2**, 485–491 (2018).

48. M. Barthelemy, *The Structure and Dynamics of Cities: Urban Data Analysis and Theoretical Modeling* (Cambridge Univ. Press, 2017).

49. L. M. A. Bettencourt, H. Samaniego, H. Youn, Professional diversity and the productivity of cities. *Sci. Rep.* **4**, 5393 (2014).

50. L. M. A. Bettencourt, Impact of changing technology on the evolution of complex informational networks. *Proc. IEEE* **102**, 1878–1891 (2014).

51. C. R. Hulten, *Growth Accounting*, NBER Working Paper 15341 (National Bureau of Economic Research, 2009).

52. A. Senhadji, Sources of economic growth: An extensive growth accounting exercise. *Staff Pap. Int. Monet. Fund* **47**, 129–157 (2000).

53. M. I. Finley, *The Ancient Economy* (Univ. of California Press, 1973).

54. A. K. Bowman, A. I. Wilson, Eds., *Quantifying the Roman Econony: Methods and Problems* (Oxford Univ. Press, 2009).

55. W. M. Jongman, in *Cambridge History of Capitalism, Volume I: From Ancient Origins to 1848*, L. Neal, J. G. Williamson, Eds. (Cambridge Univ. Press, 2014), pp. 75–100.

56. W. M. Jongman, in *Proceedings of the 23rd Annual Theoretical Roman Archaeology Conference*, H. Platts, J. Barron, C. Lundock, J. Yoo, Eds. (Oxbow Books, 2014), pp. 27–36.

57. W. M. Jongman, in *Quantifying the Greco-Roman economy and beyond*, F. Callatay, Ed. (Edipuglia, 2014).

58. I. M. Morris, *The Measure of Civilization: How Social Development Decides the Fate of Nations* (Princeton Univ. Press, 2013).

59. P. Temin, The economy of the early roman empire. *J. Econ. Perspect.* **20**, 133–151 (2006).

60. W. Scheidel, S. Friesen, The size of the economy and the distribution of income in the Roman Empire. *J. Roman Stud.* **99**, 61–91 (2009).

61. J. R. McConnell, A. I. Wilson, A. Stohl, M. M. Arienzo, N. J. Chellman, S. Eckhardt, E. M. Thompson, A. M. Pollard, J. P. Steffensen, Lead pollution recorded in Greenland ice indicates European emissions tracked plagues, wars, and imperial expansion during antiquity. *Proc. Natl. Acad. Sci. U.S.A.* **115**, 5726–5731 (2018).

62. R. C. Allen, in *Quantifying the Roman Economy*, A. Bowman, A. Wilson, Eds. (Oxford Univ. Press, 2009), pp. 327–345.

63. W. Scheidel, Ed., *The Cambridge Companion to the Roman Economy* (Cambridge Univ. Press 2012).

64. W. Scheidel, I. Morris, R. Saller, Eds., *The Cambridge Economic History of the Greco-Roman World* (Cambridge Univ. Press, 2007).

65. W. M. Jongman, J. P. A. M. Jacobs, G. M. Klein Goldewijk, Health and wealth in the Roman Empire. *Economics Hum. Biol.* **34**, 138–150 (2019).

66. A. Wilson, in *Quantifying the Greco-Roman Economy and Beyond*, F. Callatay, Ed. (Edipuglia, 2014), pp. 147–167.

67. K. Harper, *The Fate of Rome: Climate, Disease, and the End of an Empire* (Princeton Univ. Press, 2017).

68. J. W. Hanson, *An Urban Geography of the Roman World, 100 B.C. to A.D. 300* (Archaeopress, 2016).

69. A. K. Bowman, A. I. Wilson, Eds., *Settlement, Urbanization, and Population* (Oxford Univ. Press, 2011).

70. D. Mattingly, *An Imperial Possession: Britain in the Roman Empire* (Penguin Books, 2006).

71. A. Smith, M. Allen, T. Brindle, M. Fulford, *New Visions of the Countryside of Roman Britain, Volume I: The Rural Settlement of Roman Britain*, Britannia Monograph Series (Society for the Promotion of Roman Studies, 2016), **vol. 29**.

72. M. Millett, *The Romanization of Britain: An Essay in Archaeological Interpretation* (Cambridge Univ. Press, 1990).

73. M. Allen, L. Lodwick, T. Brindle, M. Fulford, A. Smith, *New Visions of the Countryside of Roman Britain, Volume II: The Rural Economy of Roman Britain*, Britannia Monograph Series (Society for the Promotion of Roman Studies, 2017), **vol. 30**.

74. M. Allen, L. Lodwick, in *New Visions of the Countryside of Roman Britain, Volume II: The Rural Economy of Roman Britain*, M. Allen, L. Lodwick, T. Brindle, M. Fulford, A. Smith, Eds. (Society for the Promotion of Roman Studies, 2017), pp. 142–177.

75. M. Pitts, *The Roman Object Revolution: Objectscapes and Intra-Cultural Connectivity in Northwest Europe* (Amsterdam Univ. Press, 2019).

76. B. Ward-Perkins, *The Fall of Rome and the End of Civilization* (Oxford Univ. Press, 2006).

77. L. Lodwick, in *New Visions of the Countryside of Roman Britain, Volume 2: The Rural Economy of Roman Britain*, M. Allen, L. Lodwick, T. Brindle, M. Fulford, A. Smith, Eds. (Society for the Promotion of Roman Studies, 2017), **vol. 30**, pp. 11–84.

78. R. Reece, Site-finds in Roman Britain. *Britannia* **26**, 179–206 (1995).

79. P. J. Casey, *Understanding Ancient Coins: An Introduction for Archaeologists and Historians* (Univ. of Oklahoma Press, 1986).

80. A. S. Robertson, in *Coins and the Archaeologist*, P. J. Casey, R. Reece, Eds. (Seaby, 1988), pp. 15–38.

81. E. Ghey, in *Coin Hoards and Hoarding in the Roman World*, J. Mairat, A. Wilson, C. Howgego, Eds. (Oxford Univ. Press, 2022), pp. 55–67.

82. R. Bland, *Coin Hoards and Hoarding in Roman Britain, AD 43-c.498*, The British Numismatic Society (Spink, 2018), **vol. special publication no. 13**.

83. J. Creighton, The supply and movement of Denarii in Roman Britain. *Britannia* **45**, 121–163 (2014).

84. P. J. Casey, in *Coins and the Archaeologist*, P. J. Casey, R. Reece, Eds. (Seaby, 1988), pp. 39–56.

85. S. Leyk, J. H. Uhl, D. S. Connor, A. E. Braswell, N. Mietkiewicz, J. K. Balch, M. Gutmann, Two centuries of settlement and urban development in the United States *Sci Adv.* **6**, eaba2937 (2020).

86. R. D. Drennan, C. A. Berrey, C. E. Peterson, *Regional Settlement Demography in Archaeology* (Eliot Werner Publications, 2015).

87. D. Perring, M. Pitts, *Alien Cities: Consumption and the Origins of Urbanism in Roman Britain*, Spoilhep Monograph Series (SpoilHeap Publications, 2013), **vol. 7**.

88. M. G. Fulford, N. Holbrook, Relevant beyond the Roman period: Approaches to the investigation, analysis and dissemination of archaeological investigations of the rural settlements and landscapes of Roman Britain. *Archaeol. J.* **175**, 214–230 (2018).

89. R. Wiseman, O. Bulik, J. Lobo, L. Lodwick, S. G. Ortman, The impact of transportation on pottery industries in Roman Britain. *Open Archaeol.* **9**, 20220286 (2023).

90. P. Tyers, *Roman Pottery in Britain* (Routledge, 2003).

91. M. D. Varien, S. G. Ortman, Accumulations research in the Southwest United States: Middle-range theory for big-picture problems. *World Archaeol.* **37**, 132–155 (2005).

92. M. D. Varien, B. J. Mills, Accumulations research: Problems and prospects for estimating site occupation span. *J. Archaeol. Method Theory* **4**, 141–191 (1997).

93. A. Y. Abul-Megd, Wealth distribution in an ancient Egyptian society. *Phys. Rev. E* **66**, 057104 (2002).

94. R. E. Blanton, *Houses and Households: A Comparative Study* (Plenum, 1994).

95. H. D. G. Maschner, R. A. Bentley, in *Complex Systems and Archaeology*, R. A. Bentley, H. D. G. Maschner, Eds. (Univ. of Utah Press, 2003), pp. 47–60.

96. J. H. Bodley, *The Power of Scale: A Global History Approach* (M. E. Sharpe, 2003).

97. K. G. Hirth, in *Prehispanic Domestic Units in Western Mesoamerica*, R. S. Santley, K. G. Hirth, Eds. (CRC Press, 1993), pp. 121–146.

98. M. D. Mitchell, *Crafting History on the Northern Plains: A Political Economy of the Heart River Region, 1400-1750* (Univ. of Arizona Press, 2013).

99. T. A. Kohler, M. E. Smith, A. Bogaard, G. M. Feinman, C. E. Peterson, A. Betzenhauser, M. C. Pailes, E. C. Stone, A. M. Prentiss, T. Dennehy, L. Ellyson, L. M. Nicholas, R. K. Faulseit, A. Styring, J. Whitlam, M. Fochesato, T. A. Foor, S. Bowles, Greater post-Neolithic wealth disparities in Eurasia than in North America and Mesoamerica. *Nature* **551**, 619–622 (2017).

100. S. G. Ortman, G. D. Coffey, Settlement scaling in middle-range societies. *Am. Antiq.* **82**, 662–682 (2017).

101. S. G. Ortman, K. E. Davis, J. Lobo, M. E. Smith, L. M. A. Bettencourt, A. Trumbo, Settlement scaling and economic change in the Central Andes. *J. Archaeol. Sci.* **73**, 94–106 (2016).

102. S. G. Ortman, J. Lobo, M. E. Smith, Cities: Complexity, theory and history. *PLOS ONE* **15**, e0243621 (2020).

103. G. Duranton, D. Puga, in *Handbook of Regional and Urban Economics*, J. V. Henderson, J.-F. Thisse, Eds. (Elsevier, 2004), **vol. 4**, pp. 2064–2117.

104. S. Angel, A. M. Blei, J. Parent, P. Lamson-Hall, N. Galarza, D. L. Civco, R. Q. Lei, K. Thom, *Atlas of Urban Expansion*: *2016 Edition Volume 1: Areas and Densities* (New York University, Urban Expansion Program, 2016).

105. D. D. Rucker, B. B. McShane, K. J. Preacher, A researcher's guide to regression, discretization, and median splits of continuous variables. *J. Consum. Psychol.* **25**, 666–678 (2015).

106. T. Brindle, in *New Visions of the Countryside of Roman Britain*, *Volume II: The Rural Economy of Roman Britain*, M. Allen, L. Lodwick, T. Brindle, M. Fulford, A. Smith, Eds. (Society for the Promotion of Roman Studies, 2017), pp. 237–280.

107. C. Haselgrove, The development of British iron-age coinage. *The Numismatic Chronicle (1966-)* **153**, 31–63 (1993).

108. D. Perring, *London in the Roman World* (Oxford Univ. Press, 2022).

109. G. de la Bedoyere, *Roman Britain: A New History* (Thames and Hudson, ed. 2, 2013).

110. W. Isard, *Location and Space-Economy: A General Theory Relating to Industrial Location, Market Areas, Land Use, and Urban Structure* (MIT Press, 1956).

111. S. Ortman, O. Bulik, R. Wiseman, J. Lobo, L. Bettencourt, L. Lodwick, Transport costs and economic change in Roman Britain. *Eur. J. Archaeol.* **27** 1–24 (2023).

112. D. Acemoglu, J. A. Robinson, *Why Nations Fail: The Origins of Power, Prosperity and Poverty* (Crown Business, 2012).

113. D. C. North, J. J. Wallis, B. R. Weingast, *Violence and Social Orders: A Conceptual Framework for Interpreting Recorded Human History* (Cambridge Univ. Press, 2009).

114. M. Koyama, J. Rubin, *How the World Became Rich: The Historical Origins of Economic Growth* (Polity Press, 2022).

115. J. B. DeLong, *Slouching Towards Utopia: An Economic History of the Twentieth Century* (Basic Books, 2022).

116. R. Fleming, *The Material Fall of Roman Britain, 300-525 CE* (Univ. of Pennsylvania Press, 2021).

117. T. Philippon, *Additive Growth*, NBER Working Paper 29950 (National Bureau of Economic Research, 2022).

118. C. Perreault, *The Quality of the Archaeological Record* (Univ. of Chicago Press, 2019).

119. M. Allen, N. Blick, T. Brindle, T. Evans, M. Fulford, N. Holbrook, L. Lodwick, J. D. Richards, A. Smith, The Rural Settlement of Roman Britain: An online resource [data-set], Archaeology Data Service (2018); https://doi.org/10.5284/1030449.

120. M. G. Fulford, L. Lodwick, A. Smith, Defended Small Towns of Roman Britain [data-set], Archaeology Data Service (2018); https://doi.org/10.5284/1046751.

121. J. Alroy, Appearance event ordination: A new biochronologic method. *Paleobiology* **20**, 191–207 (1994).

122. J.-X. Fan, S.-Z. Shen, D. H. Erwin, P. M. Sadler, N. MacLeod, Q.-M. Cheng, X.-D. Hou, J. Yang, X.-D. Wang, Y. Wang, H. Zhang, X. Chen, G.-X. Li, Y.-C. Zhang, Y.-K. Shi, D.-X. Yuan, Q. Chen, L.-N. Zhang, C. Li, Y.-Y. Zhao, A high-resolution summary of Cambrian to early triassic marine invertebrate biodiversity. *Science* **367**, 272–277 (2020).

123. S. Rippon, N. Holbrook, *Studies in the Roman and Medieval Archaeology of Exeter*, Exeter: A Place In Time (Oxbow Books, 2021), **vol. 2**.

124. S. Rippon, N. Holbrook, *Roman and Medieval Exeter and Their Hinterlands: From Isca to Excester*, Exeter: A Place in Time (Oxbow Books, 2021), **vol. 1**.

125. A. Gascoyne, D. Radford, *Colchester, Fortress of the War God: An Archaeological Assessment* (Oxbow Books, 2013).

126. S. Frere, *Verulamium Excavations, Volume I* (Society of Antiquaries, 1972).

127. M. E. Smith, T. Dennehy, A. Kamp-Whittaker, E. Colon, E. Harkness, Quantitative measures of wealth inequality in ancient central Mexican communities. *Adv. Archaeol. Pract.* **2**, 311–323 (2014).

128. M. B. Mulder, S. Bowles, T. Hertz, A. Bell, J. Beise, G. Clark, I. Fazzio, M. Gurven, K. Hill, P. L. Hooper, W. Irons, H. Kaplan, D. Leonetti, B. Low, F. Marlowe, R. McElreath, S. Naidu, D. Nolin, P. Piraino, R. Quinlan, E. Schniter, R. Sear, M. Shenk, E. A. Smith, C. von Rueden, P. Wiessner, Intergenerational wealth transmission and the dynamics of inequality in small-scale societies. *Science* **326**, 682–688 (2009).

129. P. Walton, S. Moorhead, in *The Oxford Handbook of Roman Britain*, M. Millett, L. Revell, A. Moore, Eds. (Oxford Handbooks Online, 2015).

130. K. W. Harl, *Coinage in the Roman Economy, 300 B.C. to A.D. 700* (Johns Hopkins Univ. Press, 1996).

131. D. Rathbone, in *Quantifying the Roman Economy: Methods and Problems*, A. K. Bowman, A. I. Wilson, Eds. (Oxford Univ. Press, 2009), pp. 299–327.

132. L. M. A. Bettencourt, J. Lobo, D. Strumsky, G. B. West, Urban scaling and its deviations: Revealing the structure of wealth, innovation and crime across cities. *PLOS ONE* **5**, e13541 (2010).

133. Wessex Archaeology, *Caerwent Roman Town, Monmouthshire, South Wales. Archaeological Evaluation and Assessment of Results* (Archaeology Data Service, 2009), **vol. OASIS ID 68736.01**.

134. N. Holbrook, *Cirencester: The Roman Town Defences, Public Buildings and Shops*, Cirencester Excavations (Cotswold Archaeological Trust Ltd., 1998), **vol. V**.

135. A. McWhirr, *Houses in Roman Cirencester*. Cirencester Excavations (Cirencester Excavation Committee, 1986), **vol. 3**.

136. P. Crummy, *Excavations at Culver Street, the Gilberd School, and other sites in Colchester 1971-1985*, Colchester Archaeological Reports (Colchester Archaeological Trust, 1992), **vol. 6**.

137. P. Crummy, *Excavations at Lion Walk, Balkerne Lane, and Middleborough, Colchester, Essex*, Colchester Archaeological Reports (Colchester Archaeological Trust, 1984), **vol. 3**.

138. R. P. Symonds, S. Wade, *Roman Pottery from excavations in Colchester, 1971-86*, Colchester Archaeological Reports (Colchester Archaeological Trust, 1999), **vol. 10**.

139. P. T. Bidwell, *The Legionary Bath-House and Basilica and Forum at Exeter*, Exeter Archaeological Reports (Exeter City Council, 1979), **vol. 1**.

140. A. Connor, R. Buckley, *Roman and Medieval Occupation in Causeway Lane, Leicester* (Univ. of Leicester Archaeological Services, 1999).

141. J. Creighton, R. Fry, *Silchester: Changing Visions of a Roman Town*, Britannia Monograph Series No. 28 (Society for the Promotion of Roman Studies, 2016).

142. M. G. Fulford, A. Clarke, H. Echkhardt, *Life and Labour in Late Roman Silchester: Excavations in Insula IX since 1997*, Britannia Monograph Series (Society for the Promotion of Roman Studies, 2006), **vol. 22**.

143. M. G. Fulford, *Silchester: Excavations on the Defences 1974-1980*, Britannia Monograph Series (Society for the Promotion of Roman Studies, 1984).

144. S. Frere, *Verulamium Excavations, Volume II* (Society of Antiquaries, 1983).

145. S. Frere, *Verulamium Excavations, Volume III* (Oxford University Committee for Archaeology, 1984).

146. P. Ellis, *The Roman Baths and Macellum at Wroxeter: Excavations by Graham Webster, 1955-85*, Archaeological Reports (English Heritage, 2000), **vol. 9**.

147. P. Barker, R. White, K. Pretty, H. Burd, M. Corbishley, *The Baths Basilica Wroxeter: Excavations 1966-90* (English Heritage, 1997), **vol. 8**.

148. G. Webster, *The Legionary Fortress at Wroxeter: Excavations by Graham Webster, 1955-85*, Archaeological Report (English Heritage, 2002), **vol. 19**.

149. R. H. White, C. Gaffney, V. L. Gaffney, *Wroxeter, The Cornovii an the Urban Process: Final Report on the Wroxeter Hinterland Project 1994-1997. Volume 2: Characterizing the City* (Archaeopress, 2013).

150. J. P. Bushe-Fox, *Excavations on the Site of the Roman Town at Wroxeter, Shropshire, in 1912*, Reports of the Research Committee (Society of Antiquaries of London, **1913**), **vol. 1**.

151. J. P. Bushe-Fox, *Excavations on the Site of the Roman Town at Wroxeter, Shropshire, in 1913*, Reports of the Research Committee (Society of Antiquaries of London, 1914), **vol. 2**.

152. J. P. Bushe-Fox, *Excavations on the Site of the Roman Town at Wroxeter, Shropshire, in 1914*, Reports of the Research Committee (Society of Antiquaries of London, 1916), **vol. 4**.

153. E. Durham, M. Fulford, *A Late Roman Town House and Its Environs: The Excavations of C.D. Drew and K.C. Collingwood Selby in Colliton Park, Dorchester, Dorset 1937-8*, Britannia Monograph Series (Society for the Promotion of Roman Studies, 2014), **vol. 26**.

154. P. J. Wodward, S. M. Davies, A. H. Graham, *Excavations at the Old Methodist Chapel and Greyhound Yard, Dorchester, 1981-1984*, Dorset Natural History and Archaeological Society Monograph Series (Dorset Natural History and Archaeological Society, 1993), **vol. 12**.
